# Synthesis of a Chlorella/ZnO/ZnFe_2_O_4_ green nanocomposite for preconcentration of heavy metals in food samples via ICP-OES detection

**DOI:** 10.1016/j.fochx.2026.103852

**Published:** 2026-04-24

**Authors:** Hamidreza Haghgoo Qezelje, Maryam Rajabi, Fatemeh Darabi, Yasaman Sedaghat, Amir Sajad Soleimani Kia, Alireza Asghari, Felipe de J. Silerio-Vázquez, Khalil Ahmad, Ahmad Hosseini-Bandegharaei

**Affiliations:** a Department of Chemistry, Semnan University, Semnan, Iran; b Department of Chemistry, University of Kashan, Kashan 87317-53153, Iran; c CIIDIR-Durango, Instituto Politécnico Nacional, Calle Sigma 119, Fraccionamiento 20 de Noviembre II, C. P., 34220 Durango, Mexico; d Department of Chemistry, Emerson University Multan (EUM), Multan 60000, Pakistan; e Scientific Research Center, Al-Ayen Iraqi University (AUIQ), Nasiriyah 64001, Thi-Qar, Iraq; f Department of Sustainable Engineering, Saveetha School of Engineering, SIMATS, Chennai, Tamil Nadu, 602105, India

**Keywords:** Chlorella/ZnO/ZnFe_2_O_4_ nanocomposite, Heavy metals, ICP-OES, Food samples, UA-d-μ-SPE, Green chemistry

## Abstract

A Chlorella/ZnO/ZnFe_2_O_4_ nanocomposite was synthesized and exploited for preconcentration of trace levels of heavy metals in foods, prior to measurement by ICP-OES. The synthesized nanocomposite showed outstanding properties such as high eco-degradability, cost-effectiveness, easy access to raw materials, high specific surface area, and efficient extraction recovery. It was utilized as an adsorbent in an ultrasonic-assisted D-μ-SPE procedure for preconcentration of Ni (II), Cu (II), Cd (II), Pb (II), and Hg (II) ions. Parameters were optimized and, under optimized conditions, the method attained detection limits ≤0.3 ng mL^−1^, enrichment factors ≥52.14, linear ranges varied from 0.15 to 200 ng mL^−1^ for all analytes, and RSDs ≤3.1% (*n* = 5). The proposed method was successfully implemented for determination of metal ions in a variety of food samples (13 different samples), including grains, vegetables, animal-derived products, and seafood, demonstrating high analytical applicability for trace metal monitoring in complex food systems.

## Highlights

• Chlorella/ZnO/ZnFe_2_O_4_ nanocomposite made for heavy metal preconcentration in food samples.
• Nanocomposite achieved detection limits lesser than 0.3 ng mL^−1^, with high recoveres.
• Magnetic properties enabled fast, easy separation and reusability of sorbent.
• Method works in complex food matrices including grains, vegetables, seafood.
• Sustainable, eco-friendly UA-D-μ-SPE method validated by green metrics.

## 1 Introduction

In recent decades, the fast developments of industrialization, mining, and urbanization have caused a significant escalation in pollutants within water and soil systems. Among these contaminants, heavy metals stand out as particularly harmful impurities due to their persistence and strong bio-accumulative ability in the environment and living organisms (Khatoon et al., 2024(. These metals may cause severe health risks and contribute to various diseases, once introduced into the food chain through contaminated water or soil (Ghorban et al., 2025). Recognizing such threats, international regulatory agencies have established rigorous guidelines to monitor heavy metal levels in food and other consumables. Some of the most critical heavy metals that require continuous monitoring include Ni (II), Cu (II), Cd (II), Pb (II), and Hg (II). The provisional tolerable weekly intake (PTWI) for Pb (II), Ni (II), Cu (II), and Hg (II) has been reported as 0.8, 91, 3500, and 4 μg per kilogram of body weight per week, respectively (Qezelje et al., 2025; Yap & Al-Mutairi, 2023) and the provisional tolerable monthly intake (PTMI) for Cd (II) has been reported as 25 μg per kilogram of body weight per month (World Health Organization (World Health Organization (WHO), 2021). Each of these metals influence human health in different ways. Mercury and its compounds primarily target the central nervous system, often giving rise to severe neurological and developmental impairments (Qezelje et al., 2025; Telloli et al., 2024). Nickel is associated with allergic contact dermatitis, respiratory cancers, and kidney toxicity (Genchi et al., 2020). Cadmium can damage renal function and weaken bones, as observed in the classic Itai-Itai disease, and is recognized as a human carcinogen (Rasin et al., 2025). Although copper is essential for many metabolic processes, excessive levels can induce liver and neurological disorders through oxidative stress (Witt et al., 2025). Lead interferes with both the nervous and hematopoietic systems, causing neuropsychological deficits, anemia, and hypertension, even at slight exposure (Qezelje et al., 2025). Together, such findings vouchsafe the critical need for careful monitoring of toxic heavy metals in environment and the food chain, as well as continued efforts to minimize human exposure to these potent toxins (Yap & Al-Mutairi, 2023).

The measurement of ultra-trace amounts of heavy metals in food samples is truly vital; however, the extremely low levels and complex matrices of these samples make direct quantification challenging, even with modern analytical instrumentation (Cao et al., 2023). Consequently, researchers have extensively investigated sample preparation methods that allow analyte enrichment, reduction of interference effects, and compatibility with analytical equipment. Solid phase and liquid phase extractions are among the most widely employed techniques for sample preparation (Qezelje et al., 2025). In classical SPE approaches, trace metal ions were retained on column-packed polymeric or inorganic sorbents, enabling matrix elimination and preconcentration prior to instrumental analysis (Bulut et al., 2007; Elci et al., 2000). SPE, in particular, offers remarkable merits over LLE, including higher enrichment factors and selectivity, shorter extraction times and consumption of organic solvents (Cao et al., 2023). Nonetheless, conventional SPE often requires large amounts of sorbent and sample, involves long extraction times, and is prone to column clogging. To address these weaknesses, dispersive solid-phase extraction (D-SPE) has been devised, in which adsorptive material particles are directly dispersed into the sample solution, thereby reducing extraction time. Despite all enhancements, traditional D-SPE still consumes relatively large amounts of sample and sorbent. Dispersive micro solid-phase extraction (D-μ-SPE) system has been established to overcome these challenges, significantly reducing chemical consumption. The dispersion of solid phase within sample can be obtained through various approaches. Among these, ultrasonic-assisted D-μ-SPE (UA-D-μ-SPE) has stood out as an influential way. In this approach, solid phase is uniformly dispersed throughout sample solutions using ultrasonic waves, avoiding environmental contamination and enabling rapid extraction of analytes in the shortest possible time (Bozorgzadeh et al., 2021).

The selection of an adequate sorbent is a decisive parameter that governs the overall effectiveness and success of the UA-d-μ-SPE process. The sorbent should not only exhibit selective affinity toward the target analytes but also allow rapid and efficient phase separation from the sample solution after extraction, enabling straightforward recovery of the solid phase without the need for additional centrifugation or filtration steps, while ensuring that the adsorbed species can be easily desorbed from its surface afterward (Sadegh et al., 2021). In the UA-d-μ-SPE process, sorbents are generally classified into three major groups: nanostructured materials, nanocomposites, and classical SPE materials (Azzouz et al., 2018). Among the common ones, conventional sorbents, and bio-based sorbents have received heightened attention because of their eco-friendliness and sustainability. Bio-sorbents, derived from natural sources such as microorganisms, agricultural residues, and algae, possess ample functional moieties on surface (e.g., –COOH, –OH, –NH₂) which are capable of forming strong interactions with heavy metal ions. Within this category, Chlorella species have represented great potential due to great specific surface area, biodegradability, and rich in functional groups, which enhance their affinity toward heavy metals and organic pollutants, making them suitable candidates for developing efficient and environmentally benign biosorbents (Joo et al., 2021). Traditional sorbents employed in SPE often struggle with limitations of the surface area, which reduced their adsorption potential. In contrast, nanostructured materials offer a markedly ampler specific surface area and greater availability of active sites, therefore enhancing extraction recovery and reducing equilibrium time (Azzouz et al., 2018). Examples of such nanostructures include TiO₂, CoO, ZnO, SiO₂, and ZnFe₂O₄, among others. Zinc oxide nanoparticles, in particular, have been thoroughly examined due to affordability, abundance, chemical stability, and environmental safety (Haghgoo Qezelje et al., 2025). Their extraction performance can be further optimized through strategies namely compositing or elemental doping. In recent years, magnetic ferrite nanoparticles have been increasingly employed in extraction systems because they enable facile magnet-based isolation of sorbent from sample solution, eliminating needs for centrifuging stages (Sukoviene et al., 2025). Among metal ferrites, zinc ferrite (ZnFe₂O₄) stands out owing to exceptional chemical stability, biocompatibility, magnetic behavior, and non-toxicity toward living organisms, offering both functional and environmental advantages (Sahoo et al., 2023). Nanocomposite-based sorbents can be synthesized from two or more components, with at least one existing at the nanoscale. One of the main characteristics of this category of sorbents is that they preserve the individual properties of each constituent component. Consequently, the synthesized sorbent exhibits multiple functionalities that can significantly improve the extraction of analytes and facilitate the overall extraction process (Haghgoo Qezelje et al., 2025).

The direct determination of ultra-trace heavy metals in complex food matrices by instrumental techniques such as ICP-OES presents two fundamental challenges: insufficient sensitivity for regulatory compliance and significant signal suppression or enhancement from co-existing matrix components. To achieve the requisite detection limits and ensure accurate quantification, a preconcentration and matrix simplification step is indispensable prior to analysis. To address this need, this study introduces a novel integrated method centered on a Chlorella/ZnO/ZnFe₂O₄ green nanocomposite employed in an ultrasound-assisted dispersive micro solid-phase extraction (UA-d-μ-SPE) procedure. This approach was designed to adopt materials' synergistic properties: the functional-group-rich Chlorella biomass provides high metal affinity, ZnO nanoparticles offer extensive surface area, and ZnFe₂O₄ ensures rapid magnetic isolation of adsorbent. The primary objectives were therefore to (1) synthesize and characterize this ternary nanocomposite, (2) develop and optimize a green, efficient UA-d-μ-SPE protocol for the simultaneous preconcentration of Ni (II), Cu (II), Cd (II), Pb (II), and Hg (II), and (3) rigorously validate the method's applicability for monitoring these toxic elements across a wide range of complex food samples, including seafood, vegetables, grains, and animal products, using ICP-OES detection.

## 2 Materials and methods

### 2.1 Chemicals and reagents

Stock solutions of Ni (II), Cu (II), Cd (II), Pb (II), and Hg (II) ions with concentration of 1 mg mL^−1^ were built by precise weighing and dissolving their corresponding nitrate salts (Merck, Germany) in deionized water. The working solutions were then made by stepwise dilution of stock solutions using the same solvent and were stored at 4 °C to preserve stability. Dried and pure Chlorella powder was supplied by Biogreens (Isfahan, Iran). All chemicals of analytical-grade, including nitric acid, phosphoric acid, hydrochloric acid, perchloric acid, acetic acid, stearic acid, hydrogen peroxide, anhydrous methanol, ethylenediaminetetraacetic acid (EDTA), ethylenediamine, ethanol, ammonium hydroxide, sodium hydroxide, zinc (II) acetate dihydrate, ferric (II) chloride tetrahydrate, and zinc (II) nitrate hexahydrate, were obtained from Sigma-Aldrich (St.Louis, Missouri, USA). All additional salts were of analytical grade (obtained from Merck, Germany). The standard reference materials NIST-1643f and SRM-1974c were obtained by the National Institute of Standards and Technology (Gaithersburg, Maryland, USA). The pH of prepared solutions was adjusted, when required, using 0.10 mol L^−1^ NaOH or HCl solutions.

### 2.2 Apparatus

The apparatus used for sample preparation, sorbent synthesis, characterization, and instrumental analysis are described below.

An analytical balance (AEU-120, Shimadzu, Japan) was used to weigh all substances precisely. The pH of prepared solutions was measured and adjusted using a pH meter (HI 2211, Hanna, USA). An ultrasonic bath (SW3, SONO SWISS, Switzerland), operating at 80 W and 50/60 kHz, was used to assist salt dissolution, sorbent synthesis, and extraction. A centrifuge (EBA 20, Hettich, Germany) was used to separate suspended solid particles from liquid phase.

A microwave system (MS95WCR, LG, South Korea) was utilized to synthesis of the sorbent. A mineralizer (M-9, WSL, Bytom, Poland) was employed for mineralization of samples. A high-speed rotor mill (HPM-8152, Fritsch, Germany) was applied to produce a fine powder from the samples. An electric box furnace (AFE1200L-3DSM, Atra, Qazvin, Iran), and an electric nitrogen-atmosphere tubular furnace (ATE1200L-50H55S, Atra, Qazvin, Iran) were employed for synthesis of the nanocomposite. A ball mill (MPM 2*250H-NARYA, Amin Asia Fanavarpas, Tehran, Iran), was used to grind the materials to the nanoscale and to further homogenize their components. Drying of glassware and nanomaterials was carried out in a laboratory oven (TF53, NST Lab, Iran).

The surface functional groups of fabricated sorbent were characterized, exploiting a Fourier-transform infrared (FT-IR) spectrometer (FT-IR-8400S, Shimadzu, Japan). Morphology and particle size were examined using a field-emission scanning electron microscopy (FE-SEM; MIRA3 LMU, Tescan, Czech Republic), and porosity and surface area were assessed from BET measurements (BELSORP-miniX, Microtrac, France). Crystallinity and particles' dimensions were assessed by X-ray diffraction (XRD) analysis (PW1800, Philips, Germany), and also elemental composition was appraised by the aid of an energy-dispersive X-ray spectroscopy (EDS; XL-30, Philips, Germany). Thermal stability and stabilization of Chlorella on the surface of the sorbent were investigated by thermogravimetric analysis (TGA; STA PT 1000, Linseis, Germany). The magnetic behavior of the materials was characterized using a vibrating-sample magnetometer (VSM; VSM1100, Weistron, Taiwan).

Quantitative determination of metal ions was performed under the following instrumental conditions.

Quantitative analyses of target elements were performed with an inductively coupled plasma optical emission spectrometer, ICP-OES (Genesis FEE, Spectro Genesis, Germany) equipped with a charge-coupled device (CCD) detector. Argon served concurrently as both plasma and nebulization gas. The plasma was driven at an RF power of 1.4 kW, with a coolant flow of 12.0 L min^−1^ and an auxiliary gas flow of 1.0 L min^−1^. A cross-flow nebulizer operated with a nebulizer flow of 1.0 L min^−1^ and an additional gas flow of 0.5 L min^−1^ to optimize aerosol delivery. Sample uptake was maintained at 1.2 mL min^−1^, and torch was configured in axial mode to maximize signal stability and sensitivity. Betwixt successive measurements, a 0.01 mol L^−1^ HCl wash was exploited for 30 s to become sure of the system's decontamination. The analytical wavelengths selected for Ni (II), Cu (II), Cd (II), Pb (II), and Hg (II) were 231.604 nm, 324.754 nm, 226.502 nm, 283.305 nm, and 194.227 nm, respectively.

### 2.3 Adsorbent synthesis

#### 2.3.1 Synthesis of ZnO nanoparticle

The ZnO nanoparticles were synthesized by preparing 100 mL of a 0.1 mol L^−1^ Zn(NO₃)₂·6H₂O solution. Subsequently, 0.2 mol L^−1^ NaOH and ammonium hydroxide were gradually added dropwise for adjusting the pH reach 11. The obtained mixture was then subjected to microwave irradiation (510 W) for fiftheen min, followed by sonication in an ultrasonic water bath for thirty min to enhance dispersion and crystallization. The obtained precipitate was isolated by centrifugation, thoroughly rinsed four times using pure water and twice with methanol to eliminate remaining impurities, and eventually dried in an oven (100 °C) for 2 h (Shirmahi et al., 2025).

#### 2.3.2 Synthesis of ZnFe₂O₄ nanocubes

For the synthesis of ZnFe₂O₄ nanocubes, 10 mL of a Zn(CH₃COO)₂·2H₂O with the concentration of the 0.1 mol L^−1^, and 20 mL of a FeCl₂·4H₂O with level of 0.10 molL^−1^ were built in deionized water and thoroughly mixed and stirred in a 50 mL Teflon vessel. Subsequently, 1.28 mL of liquid ethylenediamine was added. The resulting mixture was further stirred for thirty min. The Teflon vessel containing the obtained suspension was transferred within a stainless-steel autoclave. Moreover, the reaction was performed for 24 h, at 180 °C, to promote formation of ZnFe₂O₄ nanocubes. The acquired dark brown solid product was rigorously washed several times with deionized water and ethanol to retrieve remaining contaminations and, afterward, dried at 65 °C for 24 h (Fang et al., 2013).

#### 2.3.3 Synthesis of Chlorella/ZnO/ZnFe_2_O_4_ nanocomposite

For preparation of the Chlorella/ZnO/ZnFe₂O₄ nanocomposite, 3.3 g of Chlorella powder, 0.165 g of ZnO nanoparticles, and 0.55 g of ZnFe₂O₄ nanocubes were carefully weighed and mixed. To improve homogeneity and interfacial contact among the components, 0.2 g of stearic acid was introduced as a dispersing agent, which helped preclude agglomeration of components. The mixed components were applied to a ball milling (270 rpm) for 8 h to achieve uniform blending. Subsequently, the final obtained mixture was carbonized under an inert atmosphere of nitrogen (500 °C) for 2 h for facilitating the formation of the nanocomposite. The yielded product was repeatedly rinsed with deionized water to eliminate any residual impurities and finally dried in an oven (70 °C) for 5 h (Arghavani-Beydokhti et al., 2026; Shirmahi et al., 2025).

### 2.4 Preparation of food samples

#### 2.4.1 Seafoods

Sea samples (e.g., Tilapia, catfish, and shrimp) were purchased from a local market (Bandar Abbas, Iran). After separating the muscle tissue from other parts, each sample was immediately preserved on crushed ice until analysis. For preparation of each sample, 100 g of homogenized muscle tissue (wet weight) was thoroughly weighed into an iodine flask, and concentrated HNO₃ was added (50 mL). Samples were refluxed (95 ± 5 °C) for 1 h. After cooling, an exceeded 10 mL of concentrated HNO₃ was included to every sample solution, and digestion process was repeated under same conditions to become sure of completeness of digestion. When the solution volume was decreased to roughly 5 mL, 15 mL of concentrated hydrochloric acid was included, and obtained mixture was heated for 15 min. After further evaporation to about 3 mL, 30 mL of deionized water was added. Furthermore, solution was filtered through Whatman filter paper (No. 41). The filtrate was then transported to a 300 mL volumetric flask and diluted to mark with deionized water (Sen et al., 2011).

#### 2.4.2 Vegetables

Various Vegetable samples (e.g., white cabbage, carrot, radish, spinach, potato, and tomato) were supplied by a local market in Semnan (Iran). The plant samples were carefully rinsed using deionized water, then chopped into small pieces, and finally dried in an oven (45 °C). The dried plant samples were then grounded by a rotor mill, and the resulting powders were passed through a sieve (No. 18 mesh) to obtain uniform particles. The homogenized samples were stored in tightly sealed polypropylene containers and kept at 4 °C. For elemental analysis, the samples were subjected to digestion utilizing a mixture of concentrated nitric acid and hydrogen peroxide in an M-9 mineralizer. Specifically, 12 g of each plant sample was digested at 176 ± 1 °C with 20 mL of HNO_3_ and 10 mL of H_2_O_2_. The digested solutions were entirely transmitted to volumetric flask (300 mL) and diluted to volume using deionized water (Feist et al., 2008).

#### 2.4.3 Grains

Rice and corn samples were bought from grocery stores in Semnan (Iran). Initially, grain samples were oven-dried (at 65 °C). To obtain a homogeneous powdered form, each sample was separately ground using an electric grinder. From each powdered sample, 6 g was accurately weighed into polyethylene containers, and subsequently 30 mL of a hydrochloric acid–nitric acid mixture (1:3) was added. The container holding the sample was covered with a glass watch and left at room temperature (25 °C) for 24 h. Then grain samples were heated under stirring (110 ± 5 °C) for 2 h to ensure complete digestion. After cooling, the resulting solutions were transferred entirely to a volumetric flask (300 mL) and diluted to mark, using deionized water (Ebrahimi-Najafabadi et al., 2019).

#### 2.4.4 Animal-derived products

##### 2.4.4.1 Honey

Honey sample without beeswax was bought from a local grocery store in Semnan (Iran). The sample of honey was maintained for 30 min in a water bath (65 ± 1 °C) to achieve a homogeneous mixture. Subsequently, 10 g of the honey sample was carefully weighed into an Erlenmeyer flask. Afterward, 100 mL of a mixed solution of nitric acid and hydrogen peroxide in a 3:1 ratio was added. The mixture was then heated (95 ± 5 °C) until its volume was decreased to approximately 3 mL during the digestion process. After cooling, 20 mL of distilled water was added, and the solution was filtered through Whatman filter paper (No. 41). The filtrate was finally diluted to total volume of 300 mL using distilled water (Tibebe et al., 2022).

##### 2.4.4.2 Hen eggs

White eggs were provided from a local grocery store (Semnan, Iran). After thorough homogenization of the egg samples using a glass stirrer, 30 g of the sample was accurately weighed into an Erlenmeyer flask. Subsequently, 30 mL of concentrated nitric acid and 12 mL of perchloric acid were added. The container of the obtained mixture was then covered with a watch glass and heated (95 ± 5 °C) for 15 min. The digestion was continued until the sample volume was decreased to roughly 3 mL. After cooling, distilled water (20 mL) was added, and the resulting solution was filtered through Whatman filter paper (No. 41). Finally, the collected sample was diluted to a final volume of 300 mL using distilled water (Farahani et al., 2015).

### 2.5 Extraction procedure

For concurrent determination and preconcentration of studied heavy metal ions, the procedure was carried out through UA-D-μ-SPE method. For such a purpose, a 100 mL aqueous solution containing Ni (II), Cu (II), Pb (II), and Hg (II) ions (20 ng mL^−1^ each) and Cd (II) ions (10 ng mL^−1^) was prepared and its pH adjusted to 6.1. Subsequently, 10.3 mg of synthesized nanocomposite was added to prepared solutions. To enhance adsorption efficiency, the mixture of the adsorbent and sample solution was subjected in an ultrasonic water bath for 6 min. Then, magnetic sorbent phase was isolated from solutions using an external magnet.

For the desorption process, 1.9 mL of nitric acid (0.5 mol L^−1^) was then added to solid phase, and obtained mixture was sonicated for four min within an ultrasonic bath. The eluting solvent was magnetically isolated from the adsorbent and subsequently analyzed using ICP-OES.

The enrichment factor (EF) and extraction recovery (ER%) for each metal ion were computed by the aid of following equations:

$$\mathrm{EF}=\frac{C_{\mathrm{final}}}{C_{\mathrm{initial}}}$$

$$\mathrm{ER}\left(\%\right)=\frac{n_{\mathrm{final}}}{n_{\mathrm{initial}}}\times 100=\mathrm{EF}\times \frac{V_{e}}{V_{s}}\times 100$$

In these given expression, *C*_*final*_ and *C*_*initial*_ refer to concentrations of analyte in final eluting solution and initial sample solution, respectively; *n*_*final*_ and *n*_*initial*_ indicate corresponding amounts (moles) of analyte in eluent and sample phases respectively; V_s_ and *V*_*e*_ correspond to the volumes of sample and eluting solvent, respectively.

### 2.6 Experimental design methodology

To develop empirical regression models and explore the effects of key factors as well as their mutual interactions, RSM (Response Surface Methodology) was applied. In this study, a Central Composite Design (CCD) was implemented for evaluating significant variables influencing the preconcentration and determination of studying analytes. Two distinct five-level experimental matrices were employed to investigate both the adsorption and desorption processes. The Design-Expert software package (version 13.0.5.0) was applied for experimental planning and analysis.

Adsorption Stage: During this phase, the primary factors affecting both extraction and adsorption of studied analytes, i.e., sample solution pH, sorbent dosage (mg), and extraction time (min) were examined. A set of 20 experiments was performed, including 8 factorial, 6 axial, and 6 center points. The levels of the key influencing parameters and their associated experimental outcomes were summarized (Table S1).

Desorption stage: After optimizing the type of eluting solvent through the one-factor-at-a-time (OFAT) strategy, remaining variables influencing desorption stage were optimized using a multivariate strategy based on a five-level CCD. The factors examined in this design were the concentration of the eluting solvent (mol L^−1^), its volume (mL), and duration of desorption process (min). A set of 20 experiments was carried out, including 8 factorial, 6 axial, and 6 center points. The levels of the key influencing parameters and their associated experimental outcomes were summarized (Table S2).

## 3 Results and discussion

### 3.1 The structure and morphology of Chlorella/ZnO/ZnFe₂O₄ sorbent

#### 3.1.1 SEM micro-images of sorbent

**Fig. 1:**
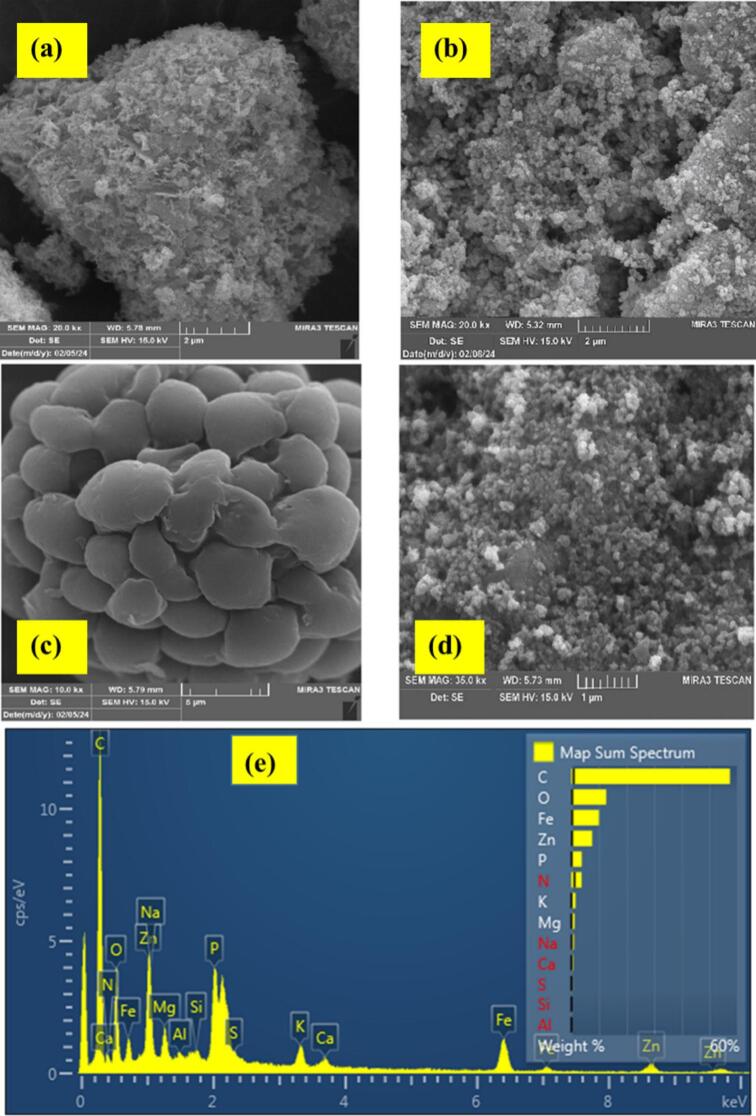
FE-SEM micro-images of (a) ZnO nanoparticles, (b) ZnFe₂O₄ nanocubes, (c) Chlorella powder, (d) synthesized Chlorella/ZnO/ZnFe₂O₄ nanocomposite, and (e) EDS spectrum of Chlorella/ZnO/ZnFe₂O₄ nanocomposite.

The morphology of the ZnO, ZnFe₂O₄, Chlorella, and Chlorella/ZnO/ZnFe₂O₄ nanocomposite was examined using FE-SEM. The FE-SEM micrograph of ZnO nanoparticles is shown in Fig. 1a. According to the image, the synthesized ZnO nanoparticles demonstrate a quasi-spherical morphology with rough and agglomerated surfaces, and their grain boundaries are clearly visible. These synthesized ZnO nanoparticles, possessing good crystallinity, high purity, and a large specific surface area, can effectively affect adsorption of heavy metal ions. The FE-SEM micro-image of ZnFe₂O₄ nanocubes is presented in Fig. 1b. As observed in this image, the particles possess a cubic morphology with a porous and relatively rough surface, which results in an uneven texture. This structural feature, along with the ferrite crystalline framework, influences the magnetic properties and consequently promotes the capability of the material to adsorb heavy metal ions. Fig. 1c illustrate FE-SEM micrograph of Chlorella powder. The image reveals that the Chlorella particles possess spherical or oval shapes and contain multiple pores and channels in their structure, which can serve as active sites for heavy metal ion adsorption. The FE-SEM image of synthesized Chlorella/ZnO/ZnFe₂O₄ nanocomposite is illustrated in Fig. 1d. The composite adsorbent displays a compact and heterogeneous structure, with cubic ZnFe₂O₄ and mostly spherical ZnO nanoparticles being fairly evenly distributed throughout carbonaceous matrix derived from Chlorella. The presence of this rough and porous texture with an ample specific surface area can enhance interfacial interaction between adsorbent and metal ions, thereby producing a highly beneficial support for preconcentration of heavy metals.

#### 3.1.2 EDS outcomes

The elemental composition of the Chlorella/ZnO/ZnFe₂O₄ nanocomposite (CZZN) was analyzed utilizing EDS. The corresponding EDS spectrum is demonstrated in Fig. 1e. According to the obtained results, the weight (%) of the constituent elements in the nanocomposite were determined as follows: C (57.47%), N (3.68%), O (12.65%), Na (0.96%), Mg (1.09%), Al (0.20%), Si (0.24%), P (3.73%), S (0.41%), K (1.46%), Ca (0.63%), Fe (9.97%), and Zn (7.53%). The high ratio of carbon indicates successful carbonization of Chlorella biomass, forming a carbonaceous matrix that serves as a supporting framework for ZnO and ZnFe₂O₄ nanoparticles. Moreover, the detectable nitrogen content implies that some nitrogen-containing functional moieties (e.g., amines) were retained on the biomass surface during carbonization, which could enhance nanocomposite's affinity for heavy metal ions. The presence of Fe and Zn peaks confirms incorporation of metal oxide phases into composite structure. Additionally, the detection of minor elements such as Na, Mg, P, and K can be assigned to inherent composition of Chlorella biomass precursor. Overall, EDS results evaluate the successful synthesis of Chlorella/ZnO/ZnFe₂O₄ nanocomposite from its constituent elements.

#### 3.1.3 TGA analysis

**Fig. 2:**
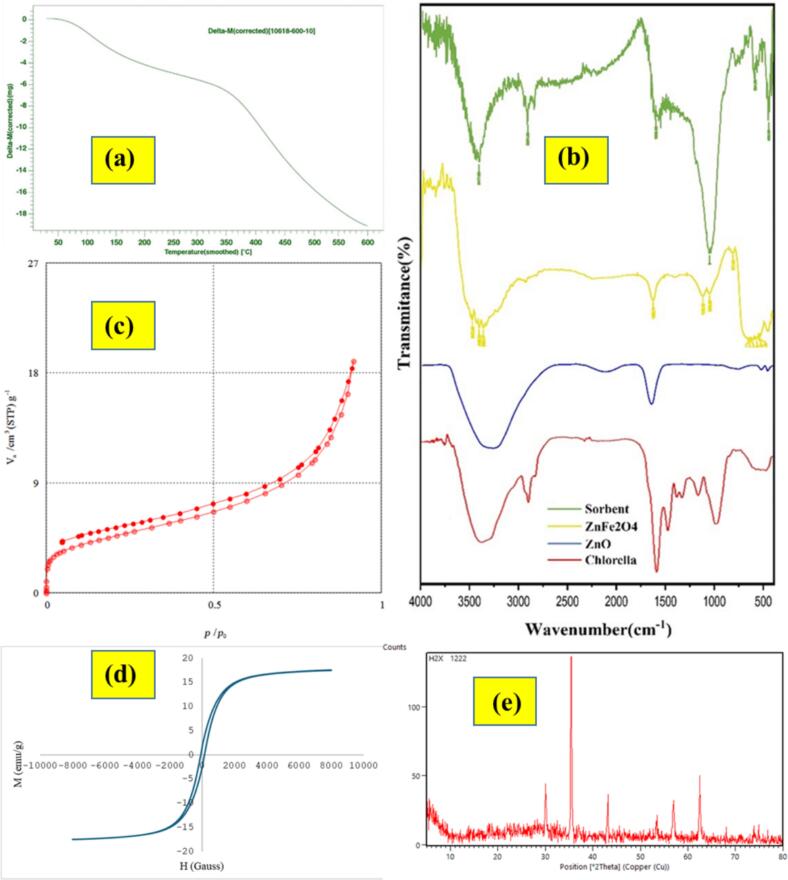
(a) Thermogravimetric analysis (TGA) of Chlorella/ZnO/ZnFe₂O₄ nanocomposite; (b) The FTIR spectra of Chlorella, ZnO, ZnFe_2_O_4_, and the synthesized nanocomposite; (c) Nitrogen sorption–desorption results of Chlorella/ZnO/ZnFe₂O₄ nanoadsorbent; (d) The magnetic spectrum pertinent to Chlorella/ZnO/ZnFe₂O₄ sorbent; and (e) XRD pattern of Chlorella/ZnO/ZnFe₂O₄ sorbent.

The thermal stability and decomposition behavior of organic constituents in the synthesized magnetic Chlorella/ZnO/ZnFe₂O₄ nanocomposite were evaluated using TGA within the temperature span of 0–600 °C in air, at a heating rate of 10 °C min^−1^. As depicted in Fig. 2a, obtained TGA curve displays a gradual weight loss occurring through three successive stages, which can be interpreted as follows. In the first stage, up to roughly 150 °C, the observed weight loss is primarily assigned to release of surface-adsorbed and physically trapped water molecules. Between 150 and 350 °C, second weight-loss stage occurs, which corresponds to decomposition of volatile organic compounds namely residual proteins and lipids originally present in the Chlorella structure. Finally, the last mass-loss stage, observed between 350 and 600 °C, is primarily associated with the thermal degradation of more stable organic constituents, such as cellulose, hemicellulose, and lignin, as well as the complete oxidation and elimination of the remaining carbonaceous residues within the nanocomposite framework.

#### 3.1.4 FT-IR spectra

The FT-IR spectrum of *Chlorella vulgaris* is shown in Fig. 2b, which vouchsafes that various functional moieties related to biomolecules (e.g., proteins, polysaccharides, lipids, and phosphate-containing compounds) are present. The absorption band observed at 1024 cm^−1^ assignable to C–O–C stretching vibration in polysaccharides, which indicates the presence of carbohydrate components (Zhao et al., 2022). An absorption peak observed at 1248 cm^−1^, can be assigned to P=O stretching mode of phosphodiester linkages within nucleic acids or other phosphate-containing biomolecules (Elsaesser et al., 2021). The band at 1383 cm^−1^ is attributed to symmetric CH₂ bending vibrations found in methyl or carboxylate (COO^−^) moieties of proteins and lipids (Elsaesser et al., 2021; Smith, 2018). The absorption band appearing at 1530 cm^−1^ is assignable to amide (II) vibration in proteins, chiefly resulting from C—N stretching and N—H bending modes. A strong peak observed at 1649 cm^−1^ represents amide (I) band, principally owing to C=O stretching of peptide linkages (Zhao et al., 2022). The band observed at 2828 cm^−1^ corresponds to symmetric and asymmetric stretching of CH₂ in lipids and carbohydrates (Smith, 2018). At approximately 3330 cm^−1^, a broad peak is observed, which corresponds to O—H stretching of water molecules and N—H stretching (amide A) of proteins (Al-Arjan, 2022). Fig. 2b also displays FT-IR spectrum of ZnO, confirming formation of ZnO nanoparticles. In span of 2710–3750 cm^−1^, a broad peak is observed, which corresponds to bending and stretching modes of O—H, and can be attributed to water molecules adsorbed on the ZnO surface (Al-Arjan, 2022). Another distinct band at 1639.7 cm^−1^ is assigned to O–H–O symmetric bending (Al-Nassar & Hussein, 2019). Furthermore, the characteristic peaks at 512.4 cm^−1^ and 448.6 cm^−1^ correspond to Zn–O–Zn stretching modes, indicating the presence of ZnO nanostructures (Al-Arjan, 2022). The FT-IR spectrum corresponding to ZnFe₂O₄ nanocubes is shown in Fig. 2b. In this spectrum, absorption peaks observed at approximately 560 cm^−1^ and 620 cm^−1^ can be attributed to Fe—O and Zn—O stretching modes in the crystal lattice (Alshehri et al., 2024). In Fig. 2b, the spectrum of the synthesized nanocomposite (Chlorella/ZnO/ZnFe₂O₄) is illustrated, which exhibits distinctive absorption bands that confirm the presence of both organic and inorganic constituents. The distinct stretching bands observed at 560–620 cm^−1^ and 448–515 cm^−1^ are assigned to Fe—O and Zn—O, respectively, verifying successful incorporation of ZnFe₂O₄ and ZnO phases within the composite lattice. The band revealed at 1645 cm^−1^ is assignable to amide (I) vibrations of polysaccharides and other organic moieties, whereas the band at 1532 cm^−1^ corresponds to amide (II) vibration. A weak band located at 1385 cm^−1^ can be ascribed to symmetric stretching mode of carboxylate groups, whereas the band at 1246 cm^−1^ is attributable to P=O stretching vibration of phosphate functionalities. Moreover, the stretching vibration peak observed at 1026 cm^−1^ is assigned to C–O–C of polysaccharides. The broad absorption band centered around 3335 cm^−1^ is indicative of N—H and O—H stretching vibrations, stemming back from hydroxyl and amino groups that are present in both Chlorella-derived biomolecules and the surface hydroxyls of the oxides. Collectively, these spectral features confirm the structural and formation integrity of the Chlorella/ZnO/ZnFe₂O₄ nanocomposite, as well as the effective integration of biogenic and inorganic components within the hybrid material.

#### 3.1.5 BET analysis

The N₂ adsorption–desorption isotherm of synthesized nanocomposite, shown in Fig. 2c, exhibits a pronounced hysteresis loop at relative pressures (p/p₀) > 0.4, indicating its mesoporous characteristics. At low relative pressures, the gradual uptake of nitrogen indicates monolayer formation, whereas the sharp increase at relatively high pressures is ascribed to capillary condensation within the mesopores.

#### 3.1.6 VSM analysis

As illustrated in Fig. 2d, the magnetic behavior of Chlorella/ZnO/ZnFe₂O₄ nanocomposite was evaluated using VSM at room temperature (25 °C). The M–H curve (±10 kOe) exhibits a narrow S-shaped hysteresis loop with a saturation magnetization (Ms) of about 18 emu g^−1^, coercivity (Hc) of approximately 120 G, and remanent magnetization (Mr) of 0.6 emu g^−1^, indicating a superparamagnetic-like nature typical of nanosized ferrite particles. The relatively high Ms. value confirms successful incorporation of ZnFe₂O₄ while very low Mr. and Hc demonstrate the nanocomposite's excellent magnetic responsiveness, which enables quick separation and easy redispersion after field removal. The slight reduction in Ms. compared with pure ZnFe₂O₄ arises from presence of non-magnetic ZnO and the Chlorella matrix, which dilute the overall magnetization and prevent magnetic aggregation. Therefore, nanocomposite exhibits suitable magnetic properties for efficient magnetic separation and reusability in adsorption applications.

#### 3.1.7 XRD pattern

The XRD pattern of CZZN is displayed in Fig. 2e, which confirms the coexistence of hexagonal wurtzite ZnO and cubic spinel ZnFe₂O₄ crystalline phases. The diffraction peaks at 2θ = 31.7°, 34.4°, 36.2°, 47.5°, 56.6°, and 62.8° are assigned to the (100), (002), (101), (102), (110), and (103) planes of ZnO (JCPDS No. 36–1451) (Shirmahi et al., 2025), while those near 30.4°, 35.7°, 43.3°, 53.6°, 56.8°, and 62.9° are attributable to (220), (311), (400), (422), (511), and (440) planes of ZnFe₂O₄ (JCPDS No. 01–077-0011) (Alshehri et al., 2024). A broad weak hump below 15° indicates the presence of the amorphous biogenic matrix derived from Chlorella. The lack of additional impurity peaks indicates the successful integration of ZnO and ZnFe₂O₄ nanoparticles within the composite structure.

#### 3.1.8 PZC point

To find PZC (point of zero charge) of synthesized sorbent, 50 mL of 0.01% (*w*/*v*) sodium chloride solutions were prepared, and their pH were adjusted to 2, 4, 6, 8, 10, and 12. Subsequently, 0.02 g of CZZN was introduced into each solution, and resulting mixtures were stirred for around 48 h to reach equilibrium. After this period, the final pH values of all suspensions were measured, and corresponding results are displayed in Fig. S1a, and b. As shown, the isoelectric point was found to occur at approximately pH 5.9, at which CZZN surface becomes electrically neutral. This indicates that at pH values below 5.9, sorbent surface carries a positive charge. However, at higher pH values (>5.9), it becomes negatively charged, which is favorable for the adsorption of metal cations. Since most metal ions exist as cationic species in the aqueous media within the pH range of 5–8, a ZPC value of approximately 5.9 provides favorable conditions for their effective adsorption under nearly neutral environments. This is practically and environmentally useful, as it minimizes the need for harsh pH adjustment. Therefore, based on the determined ZPC value, it can be inferred that the optimal operational range for cation adsorption lies at pH values above 5.9, where the negatively charged surface promotes strong electrostatic interactions with positively charged ions.

### 3.2 Effect of each component of CZZN on the extraction recovery

To assess the effect of each component of the nanocomposite on extraction recoveries (%) of Ni (II), Cu (II), Cd (II), Pb (II), and Hg (II), an aqueous solution containing a mixture of these analytes was subjected to UA-D-μ-SPE procedure using Chlorella, ZnO, ZnFe₂O₄, and the synthesized Chlorella/ZnO/ZnFe₂O₄ nanocomposite at three different pH values (3, 6, and 9). The preconcentration and subsequent determination of the target metal ions were performed using ICP-OES, and obtained findings are illustrated in Fig. S2. The outcomes demonstrate that the combination of all components in the nanocomposite formulation leads to a higher extraction recovery (%) for each analyte compared to using any individual component alone. This finding confirms the successful synthesis of a nanocomposite with superior capability for preconcentration and determination of studied analytes.

### 3.3 Statistical evaluations

The ANOVA (analysis of variance) results for adsorption stage of the studied ions (e.g., Ni (II), Cu (II), Cd (II), Pb (II), and Hg (II)) by the use of Chlorella/ZnO/ZnFe₂O₄ through UA-D-μ-SPE technique is presented in Table S3. *P*-values were exploited to evaluate the relevance of the proposed model. Since the calculated *p*-values were inferior than 0.05 (at 95% confidence level), null hypothesis was found to be invalid, confirming that the model has a meaningful effect. Furthermore, the lack-of-fit test *p*-value exceeded 0.05, confirming that unexplained variation was not statistically noteworthy at confidence level of 95%. Such results demonstrate the adequacy and reliability of developed model. Moreover, the final regression equations in coded form for the five analytes are given below, where Y₁, Y₂, Y₃, Y₄, and Y₅ indicate the extraction recovery (%) of Ni (II), Cu (II), Cd (II), Pb (II), and Hg (II) in the adsorption stage, respectively.

Y_1_ = 88.12 + 1.23 A + 1.71 B + 1.57 C + 0.8375 AB + 0.0125 AC + 0.0875 BC – 12.73 A^2^ – 6.64 B^2^ – 6.38 C^2^

Y_2_ = 91.43 + 1.43 A + 1.55 B + 1.58 C + 0.8500 AB + 0.1500 AC + 0.0250 BC – 12.65 A^2^ – 7.02 B^2^ – 6.56 C^2^

Y_3_ = 89.33 + 1.39 A + 1.68 B + 1.57 C + 0.6750 AB – 0.0251 AC + 0.1250 BC – 12.62 A^2^ – 6.70 B^2^ – 6.27 C^2^

Y_4_ = 90.40 + 1.42 A + 1.67 B + 1.53 C + 0.7000 AB – 0.2750 AC – 0.0500 BC – 12.62 A^2^ – 6.91 B^2^ – 6.45 C^2^

Y_5_ = 87.02 + 1.28 A + 1.76 B + 1.6 C + 0.8875 AB + 0.0125 AC + 0.1375 BC – 12.59 A^2^ – 6.55 B^2^ – 6.44 C^2^

Table S3 also presents the R^2^, adjusted R^2^, and predicted R^2^ values for the investigated ions. These findings confirm that proposed model is robust, accurate, and capable of making precise predictions. Moreover, inspection of Fig. S3–S7, corresponding to Ni (II), Cu (II), Cd (II), Pb (II), and Hg (II), reveals that the data approximately follow a normal distribution, show good agreement betwixt experimental and predicted values, and show no systematic errors. Collectively, these observations support that the proposed model is robust and trustworthy.

The ANOVA results for desorption studied ions (e.g., Ni (II), Cu (II), Cd (II), Pb (II), and Hg (II)) and their detachments from Chlorella/ZnO/ZnFe₂O₄ via the UA-d-μ-SPE technique is presented in Table S4. P-values were exploited to evaluate relevance of the proposed model. Since calculated p-values were inferior than 0.05 (at confidence level of 95%), null hypothesis was ruled out, confirming statistical significance of model. Moreover, the lack-of-fit test p-value exceeded 0.05, confirming that unexplained variation was not statistically noteworthy at the confidence level of 95%. Such results demonstrate adequacy and reliability of the developed model. The final regression equations in coded form for the five analytes are presented below, where Y_6_, Y_7_, Y_8_, Y_9_, and Y_10_ represent the extraction recovery (%) of Ni (II), Cu (II), Cd (II), Pb (II), and Hg (II) in the desorption stage, respectively.

Y_6_ = 98.75 + 4.24 A + 1.86 B + 3.83 C – 3.06 AB + 1.01 AC + 1.37 BC – 4.53 A^2^ – 13.76 B^2^ – 7.15 C^2^

Y_7_ = 99.63 + 4.18 A + 1.78 B + 3.77 C – 2.75 AB + 1.01 AC + 1.29 BC – 4.58 A^2^ – 13.78 B^2^ – 7.04 C^2^

Y_8_ = 98.93 + 4.23 A + 1.89 B + 3.80 C – 3.03 AB + 1.01 AC + 1.34 BC – 4.50 A^2^ – 13.80 B^2^ – 7.13 C^2^

Y_9_ = 99.29 + 4.14 A + 1.65 B + 3.81 C – 2.70 AB + 1.05 AC + 1.27 BC – 4.43 A^2^ – 13.79 B^2^ – 7.22 C^2^

Y_10_ = 98.51 + 4.25 A + 1.87 B + 3.84 C – 3.05 AB + 0.9988 AC + 1.30 BC – 4.53 A^2^ – 13.75 B^2^ – 7.17 C^2^

Table S4 also presents the R^2^, adjusted R^2^, and predicted R^2^ values for the investigated ions. These findings confirm that the proposed model is robust, accurate, and capable of making precise predictions. Furthermore, inspection of Fig. S8–S12, corresponding to Ni (II), Cu (II), Cd (II), Pb (II), and Hg (II), reveals that the data approximately follow a normal distribution, show high coply betwixt experimental and predicted values, and show no systematic errors. Collectively, these observations confirm the model reliability.

### 3.4 Optimization

#### 3.4.1 Optimizing the adsorption by CCD

**Fig. 3:**
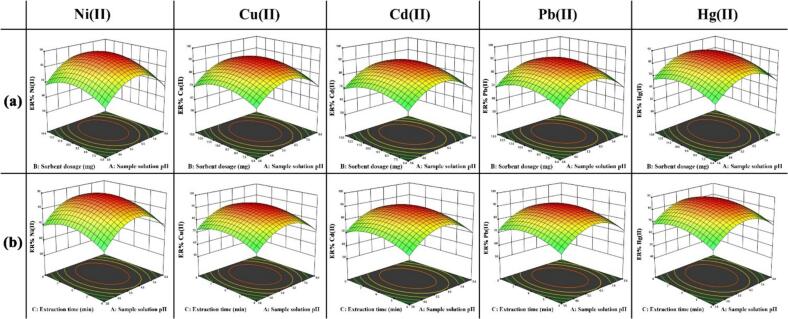
Interaction influence of (a) sorbent dosage and sample solution pH, and (b) extraction duration and sample solution pH.

Fig. 3a illustrates the interactions betwixt sample solution pH and adsorbent dosage on the extraction recovery of the studied metal ions. It is observed that at low adsorbent dosages (e.g., 4 mg), the extraction recoveries of all analytes were relatively low, typically below about 70% for all analytes. By increasing the adsorbent dosage up to about 10 mg, the extraction recovery increased markedly and approached its maximum, reaching above 87% under the optimized conditions. This behavior can be assigned to limited number of active adsorption sites at low adsorbent dosages, which restricts complete analyte extraction. As the adsorbent amount increases, more active sites are available, which improves the extraction recovery. At higher loadings, however, particle agglomeration and the consequent reduction of effective surface area and accessible active sites occur and reduce specific surface area, leading to a drop in extraction recovery to about 58–74% for the different ions. The effect of pH is also evident in Fig. 3a. At strongly acidic pH values (pH ≈ 2–4), the extraction recoveries of all ions remain low in the range of 49–66%, while it gradually increases with pH and reaches its maximum around pH 6. In this region, recoveries close to the optimum values (87–92%) are obtained. Beyond this point, the extraction recovery declines with further pH elevation. This trend is consistent with the surface charge of the adsorbent. Underneath acidic conditions, the adsorbent surface carries a positive charge (as inferred from its point of zero charge (pHpzc)), which results in electrostatic repulsion toward the cationic analytes, thereby suppressing adsorption. As the pH rises to approximately 6, the adsorbent surface becomes negatively charged, strengthening the electrostatic attraction with the metal cations and enhancing adsorption. However, at excessively high pH values (pH > 6), a slight decline in recovery is observed around 52–72%. This behavior can be related to competition from excess hydroxide ions and possible hydrolysis/precipitation of metal ions, both of which reduce the effective interaction between the analytes and the adsorbent.

The interaction effect between extraction time and the sample solution pH is illustrated in Fig. 3b. As observed, at short adsorption times (≤ 2–4 min), the extraction recovery of the analytes is relatively low (below 70% for most ions); indicating insufficient contact time to reach equilibrium. Increasing the adsorption time up to about 6 min markedly improves the extraction. Nevertheless, at prolonged adsorption times, the intense ultrasonic energy may cause desorption of previously adsorbed ions from surface of adsorbent, thereby reducing the overall extraction recovery of the studied metal ions.

Based on the optimization outcomes obtained from function desirability, the most favorable conditions for adsorption of the investigated metal ions were determined to be an adsorbent dosage of 10.3 mg, a solution pH of 6.1, and an adsorption duration of 6.0 min. Under these conditions, extraction recoveries for Ni (II), Cu (II), Cd (II), Pb (II), and Hg (II) were 88.34%, 91.64%, 89.56%, 90.62%, and 87.26%, respectively, corresponding to a global desirability value of 0.981. These results demonstrate that the CCD-based optimization provides a robust compromise between high extraction efficiency and minimal adsorbent consumption and processing time.

#### 3.4.2 Eluting solvent type

**Fig. 4:**
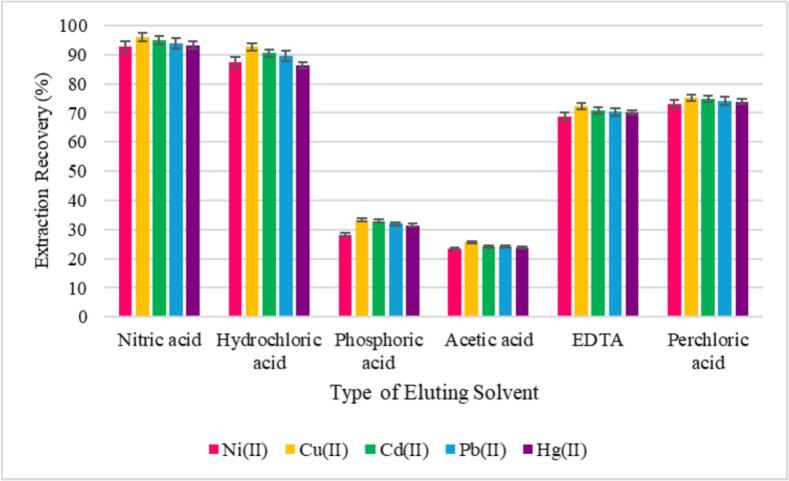
Optimization of eluting solvent type underneath conditions 100 mL of aqueous solution containing Ni (II), Pb (II), Cu (II), and Hg (II) ions (each 20 ng mL^−1^) and Cd (II) ions (10 ng mL^−1^); sorbent dosage 10.3 mg; extraction time 6 min; sample solution pH 6.1; 2 mL of each solvent (0.3 mol L^−1^) as eluting solvent; and desorption duration 3 min.

At this stage, influence of the eluent type was evaluated. The eluting solvent bears a vital role in desorption of heavy metal ions previously retained on the sorbent surface. An efficient eluting solvent should be capable of disrupting interactions betwixt analytes and active sites, allowing their complete release into the solution phase. To assess this, several eluents, including HNO₃, HCl, H₃PO₄, CH₃COOH, EDTA, and HClO₄ (each at 0.3 mol L^−1^), were tested. As depicted in Fig. 4, HNO₃ showed highest extraction recovery (%) of the studied metal ions. This superior efficiency is chiefly assigned to its strong proton-donating ability and its oxidizing nature, which effectively disrupts metal–adsorbent interactions and promotes complete desorption of the target ions.

#### 3.4.3 Optimizing desorption

**Fig. 5:**
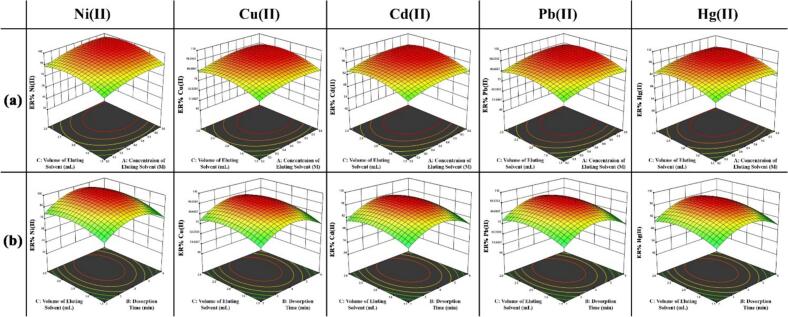
The interaction effect of (a) volume of eluting solvent and concentration of eluting solvent, and (b), volume of eluting solvent and desorption time.

Fig. 5a illustrates interaction effect between volume and concentration of the eluting solvent on the extraction recovery of assessed metal ions. As observed, at low eluting solvent's volumes (≈ 0.9–1.3 mL), extraction recoveries of analytes are clearly insufficient, with values below 75% for all analytes. This behavior indicates that the available eluent is not enough to completely strip the ions from the sorbent surface. Increasing the volume to about 1.8–2.0 mL leads to a sharp increase in recoveries. Since the extraction recovery remained constant at higher volumes, larger volumes were avoided to prevent a decline in the enrichment factor. A similar behavior is observed for the eluent concentration. At low acid concentrations (< 0.4 mol L^−1^ HNO₃), the hydronium ion concentration is insufficient to efficiently break the interactions between the metal ions and the adsorbent active sites, and the extraction recoveries remain below 80%. Increasing the concentration of eluting solvent up to about 0.5 mol L^−1^ markedly improves desorption, yielding almost quantitative recoveries (≈98–100%) for all analytes. Further increases in acid concentration do not provide any significant improvement in recovery, confirming that 0.5 mol L^−1^ is adequate to achieve complete desorption. Considering both efficiency and environmental aspects, higher acid concentrations were not employed due to unnecessary acid consumption and potential environmental hazards, while providing no analytical benefit.

The interaction between desorption time and eluting volume (Fig. 5b) further supports these conclusions. As shown, at short desorption times (≈1–2 min), the extraction recoveries were relatively low, 57–83%, reflecting insufficient contact time for the eluent to fully disrupt the metal–adsorbent interactions. Increasing the time to approximately 4 min improved the recovery for all analytes. Prolonging the desorption time beyond 4 min leads to a slight decrease in recovery, likely due to re-adsorption phenomena or redistribution of the desorbed ions on the sorbent surface.

Based on the optimization outcomes obtained from function of desirability, the most favorable conditions for desorption of assessed metal ions were determined to be an eluting solvent concentration of 0.5 mol L^−1^, an eluting solvent volume of 1.9 mL, and a desorption duration of 4 min. The extraction recoveries for Ni (II), Cu (II), Cd (II), Pb (II), and Hg (II) were 99.3%, 100.2%, 99.49%, 99.83%, and 99.07%, respectively. The corresponding desirability value (1.000) confirms the excellent overall optimization performance of the desorption step.

### 3.5 Figures of merits

The critical advantage of the developed UA-d-μ-SPE method lies in its significant enhancement of sensitivity compared to direct analysis.

Under the optimized conditions, figures of merit of UA-d-μ-SPE method for the studied analytes are illustrated in Table 1. For Ni (II), Cu (II), Cd (II), Pb (II), and Hg (II), limits of detection (LODs) were 0.15, 0.1, 0.04, 0.30, and 0.09 ngmL^−1^, the linear dynamic ranges (LDRs) were 0.50–150.00, 0.35–120.00, 0.15–80.00, 1.00–200.00, and 0.30–100.00 ng mL^−1^, and enrichment factors (EFs) were 52.26, 52.74, 52.36, 52.54, and 52.14, respectively. The calibration curves related to the studied cations are presented in Fig. S13-S17. At the concentration level of 5 ng mL^−1^, relative standard deviation (RSD) for the studied analytes was determined. Based on the results, the RSDs for Ni (II), Cu (II), Cd (II), Pb (II), and Hg (II) are 3.0, 2.9, 2.8, 3.0, and 3.1%, respectively. Additionally, according to the results shown in Fig. S18, without a significant drop in the extraction percentage, the synthesized sorbent is capable of extracting the studied metal ions up to 9 times.

**Table 1:** Merit's figures of UA-D-μ-SPE for the studied heavy metals.

Parameter	Ni (II)	Cu (II)	Cd (II)	Pb (II)	Hg (II)
ER^a^%	99.30	100.20	99.49	99.83	99.07
EF^b^	52.26	52.74	52.36	52.54	52.14
LDR^c^	0.50–150.0	0.35–120.0	0.15–80.0	1.00–200.00	0.30–100.0
LOD^d^	0.15	0.10	0.04	0.30	0.09
Intra-day precision (RSD^e^%)	3.0	2.9	2.8	3.0	3.1
Inter-day precision (RSD%)	3.7	3.5	3.4	3.5	3.8

a Extraction Recovery.

b Enrichment Factor.

c Linear Dynamic Range (ng mL^−1^).

d Limit of Detection (ng mL^−1^).

e Relative Standard Deviation (5 ng mL^−1^), (*n* = 5).

To demonstrate the advantage of the developed UA-d-μ-SPE method, the LOD values obtained from direct determination of the targeted metal ions by ICP-OES were compared with those of UA-d-μ-SPE method. The direct LOD values were achieved by direct aspiration of samples with suitable concentrations of each metal ions into the ICP-OES, without preconcentration. The results revealed that the preconcentration step lowers the detection limits by approximately more than 40 folds, for all target analytes. For instance, the LOD for Cd (II) was improved from 1.98 ng mL^−1^ (direct analysis) to 0.04 ng mL^−1^, i.e., a nearly 50-fold enhancement. This substantial improvement validates the necessity of the preconcentration step to meet the stringent sensitivity requirements for monitoring ultra-trace heavy metals in food samples, at regulatory levels. Indeed, considering the obtained statistical parameters, the proposed method can be regarded as one of effective techniques for determination and preconcentration of the investigated metal cations at ultra-trace level.

### 3.6 Interference effects

The coexistence of different ions in real samples necessitates evaluating selectivity of the proposed method to ensure the accurate quantification of the investigated ions. Therefore, the potential effects of co-existing ions that could interfere with extraction or determination processes were systematically investigated, and the findings are presented in Table 2. As illustrated, the extraction recovery of the metal ions remained unaffected by presence of Group I and Group II cations from the periodic table, even at concentrations several hundred times higher than those of the analytes. Similarly, halide ions and other common anions typically found in environmental matrices did not cause any noticeable interference. Moreover, among the investigated transition metals, zinc exhibited the highest potential for interference; however, even at concentrations up to 120 times greater than those of the analytes, its effect remained insignificant, and the analytical results were still acceptable. Additional experiments were conducted to assess impacts of multiple-fold increases in the concentration of each ion on the measurement accuracy of other analytes. The obtained results confirmed the satisfactory performance of the adsorbent, demonstrating its strong selectivity and capability for simultaneous extraction of studied metal cations, even in complex media containing high levels of coexisting species.

**Table 2:** Assessment of effects of foreign ions on recovery of studied heavy metal ions underneath the best circumstances.

Ion	Ratio interfering with ions to analytes^a^	Added as	Recovery% (RSD%)				
			Ni (II)	Cu (II)	Cd (II)	Pb (II)	Hg (II)
Na^+^	1000	NaCl	97.33 (1.4)	98.64 (1.3)	97.52 (1.3)	97.64 (1.5)	96.84 (1.6)
K^+^	1000	KCl	96.99 (1.3)	98.35 (1.1)	97.41 (1.3)	97.16 (1.4)	96.98 (1.5)
Mg^2+^	800	Mg(NO_3_)_2_.6H_2_O	96.57 (1.1)	97.38 (1.2)	96.92 (1.4)	96.39 (1.3)	96.44 (1.4)
Ca^2+^	800	CaCO_3_	96.46 (1.3)	96.88 (1.3)	96.75 (1.5)	96.26 (1.2)	96.37 (1.6)
Mn^2+^	500	Mn(NO_3_)_2_.6H_2_O	96.02 (1.5)	96.43 (1.4)	96.27 (1.4)	96.12 (1.3)	96.11 (1.4)
Co^2+^	500	Co(NO_3_)_2_.6H_2_O	96.31 (1.4)	96.52 (1.5)	96.14 (1.1)	96.61 (1.2)	96.34 (1.5)
Zn^2+^	120	Zn(NO_3_)_2_.6H_2_O	96.45 (1.5)	96.37 (1.6)	94.82 (1.5)	96.36 (1.3)	96.04 (1.6)
Fe^3+^	500	Fe(NO_3_)_3_.6H_2_O	96.48 (1.2)	96.75 (1.3)	96.64 (1.4)	96.31 (1.2)	96.27 (1.7)
Al^3+^	500	Al(NO_3_)_2_.6H_2_O	97.45 (1.3)	97.62 (1.2)	97.57 (1.6)	97.61 (1.3)	97.38 (1.4)
As^3+^	500	As_2_O_3_	97.31 (1.3)	97.48 (1.3)	97.68 (1.5)	97.16 (1.3)	97.05 (1.5)
F^−^	1000	NaF	96.84 (1.3)	98.12 (1.1)	97.23 (1.2)	97.43 (1.4)	96.71 (1.7)
Cl^−^	1000	NaCl	97.33 (1.4)	98.64 (1.3)	97.52 (1.3)	97.64 (1.5)	96.84 (1.6)
NO_3_^−^	1000	NaNO_3_	97.41 (1.3)	98.53 (1.3)	97.36 (1.6)	97.25 (1.3)	97.01 (1.7)
CO_3_^2−^	800	CaCO_3_	96.46 (1.3)	96.88 (1.3)	96.75 (1.5)	96.26 (1.2)	96.37 (1.6)
CH_3_COO^−^	500	CH_3_COONa	96.12 (1.1)	96.51 (1.5)	96.13 (1.5)	96.07 (1.3)	96.10 (1.7)
SO_4_^2−^	500	Na_2_SO_4_	95.76 (1.5)	96.13 (1.2)	95.88 (1.3)	95.71 (1.5)	95.64 (1.6)
PO_4_^3−^	500	Na_3_PO_4_	96.32 (1.2)	96.99 (1.5)	96.61 (1.3)	96.56 (1.4)	96.41 (1.5)
Ni^2+^	80	Ni(NO_3_)_2_.6H_2_O	–	95.51 (1.7)	95.30 (1.4)	95.71 (1.6)	95.37 (1.7)
Cu^2+^	70	Cu(NO₃)₂·3H₂O	94.82 (1.6)	–	95.49 (1.4)	96.03 (1.5)	95.57 (1.7)
Cd^2+^	80	Cd(NO₃)₂·4H₂O	95.69 (1.3)	96.59 (1.5)	–	95.52 (1.6)	94.46 (1.6)
Pb^2+^	80	Pb(NO₃)₂	95.77 (1.4)	96.33 (1.4)	95.17 (1.7)	–	94.58 (1.7)
Hg^2+^	85	Hg(NO₃)₂	95.96 (1.5)	96.29 (1.4)	95.01 (1.6)	95.50 (1.6)	–

a Concentration of each analytes are 20.0 ng mL^−1^ (*n* = 3).

### 3.7 Evaluation of real sample analysis

Direct quantification of investigated heavy metals, (including Ni (II), Cu (II), Cd (II), Pb (II), and Hg (II) ions), in complex food samples (e.g., seafood, vegetables, grains, and animal-derived products) is often unfeasible due to their intricate composition and the trace-level concentrations of the analytes of interest. To address these challenges, a Chlorella/ZnO/ZnFe₂O₄ nanocomposite was prepared and successfully applied as a sorbent in UA-d-μ-SPE procedure. The applicabilities of developed method was validated on miscellaneous food samples, which were analyzed under the optimized conditions outlined in previous sections. The accuracy and influence of the sample matrix of the developed method were appraised by measuring relative recoveries through the standard addition method at two levels (5 and 10 ng mL^−1^). This approach allowed for evaluating influence of each sample matrix on relative recovery (RR%) of analytes. The RR% values were calculated using Eq. (3):

$$\mathrm{RR}\%=\frac{\left(C_{\mathrm{found}}-C_{\mathrm{initial}}\right)}{C_{\mathrm{added}}}\times 100$$

where *C*_*found*_ is the measured concentration after standard addition, *C*_*initial*_ represents the initial concentration of the heavy metal ions in the sample prior to spiking, and *C*_*added*_ denotes the concentration of the standard added.

Table 3 summarizes the RR% values for all analyzed samples, which ranged from 95.1% to 103.9%, with RSD% ≤ 1.5% (*n* = 3). These outcomes demonstrate that developed method enables reliable quantification of trace-level metal cations in the complex food matrices, with negligible interference from matrix components.

**Table 3:** Heavy metal ions' relative recovery (RR%) in actual samples using the standard addition procedure under ideal circumstances.

Real Sample	C_added_	Ni (II)		Cu (II)		Cd (II)		Pb (II)		Hg (II)	
		C_found_	RR% (RSD%)	C_found_	RR% (RSD%)	C_found_	RR% (RSD%)	C_found_	RR% (RSD%)	C_found_	RR% (RSD%)
Catfish	zero	174.3	-a	2690.2	–	15.6	–	270.5	–	8.2	–
	5	179.2	97.8 (1.3%)	2695.0	96.2 (1.4%)	20.5	96.9 (1.3%)	275.4	97.1 (1.2%)	13.0	96.8 (1.4%)
	10	184.2	98.6 (1.2%)	2700.0	97.8 (1.2%)	25.4	97.5 (1.2%)	280.3	98.4 (1.1%)	18.0	97.9 (1.2%)
Tilapia	zero	215.7	–	2860.3	–	30.1	–	220.9	–	6.6	–
	5	220.5	96.7 (1.4%)	2865.1	96.6 (1.4%)	35.0	97.2 (1.2%)	225.7	96.2 (1.3%)	11.4	96.1 (1.4%)
	10	225.5	97.9 (1.3%)	2870.0	97.0 (1.3%)	40.0	98.8 (1.1%)	230.6	96.9 (1.2%)	16.3	97.3 (1.3%)
Shrimp	zero	364.0	–	1330.1	–	13.2	–	432.6	–	3.0	–
	5	368.9	97.1 (1.3%)	1334.9	96.9 (1.4%)	18.1	97.6 (1.2%)	437.5	97.4 (1.2%)	7.9	97.7 (1.3%)
	10	373.8	97.9 (1.2%)	1339.9	97.8 (1.2%)	23.0	98.3 (1.1%)	442.4	98.1 (1.1%)	12.8	98.2 (1.2%)
White cabbage	zero	465.0	–	1121.0	–	66.0	–	477.1	–	8.3	–
	5	470.1	102.7 (1.4%)	1126.1	103.7 (1.5%)	71.2	103.6 (1.3%)	482.2	102.8 (1.3%)	13.5	103.1 (1.5%)
	10	475.1	101.2 (1.1%)	1131.1	102.4 (1.3%)	76.3	102.5 (1.2%)	487.3	101.9 (1.2%)	18.5	102.3 (1.3%)
Carrot	zero	156.3	–	1211.0	–	110.2	–	189.5	–	0.3	–
	5	161.4	102.4 (1.3%)	1216.2	102.9 (1.4%)	115.4	103.9 (1.3%)	194.7	103.7 (1.4%)	5.5	103.6 (1.5%)
	10	166.5	101.8 (1.2%)	1221.2	101.6 (1.3%)	120.5	102.8 (1.2%)	199.8	103.1 (1.3%)	10.5	102.2 (1.3%)
Radish	zero	184.7	–	195.3	–	52.0	–	410.2	–	19.8	–
	5	189.8	102.9 (1.4%)	200.5	103.7 (1.5%)	57.1	102.8 (1.2%)	415.4	103.5 (1.4%)	25.0	103.2 (1.4%)
	10	194.9	102.2 (1.3%)	205.6	102.6 (1.4%)	62.1	101.3 (1.1%)	420.4	102.4 (1.3%)	30.0	102.1 (1.3%)
Spinach	zero	280.7	–	610.9	–	120.5	–	380.0	–	0.34	–
	5	285.5	96.1 (1.4%)	615.7	95.5 (1.4%)	125.3	96.1 (1.3%)	384.8	95.2 (1.4%)	5.1	95.6 (1.5%)
	10	290.4	97.2 (1.3%)	620.5	96.4 (1.3%)	130.2	96.8 (1.2%)	389.6	96.1 (1.3%)	10.0	96.3 (1.4%)
Potato	zero	860.7	–	4871.0	–	190.3	–	1120.9	–	0.45	–
	5	865.6	97.7 (1.3%)	4875.2	98.2 (1.2%)	195.2	98.4 (1.1%)	1125.8	97.8 (1.2%)	5.40	98.3 (1.2%)
	10	870.5	98.3 (1.2%)	4880.9	99.6 (1.0%)	200.2	99.1 (1.0%)	1130.8	98.5 (1.1%)	10.40	99.4 (1.0%)
Tomato	zero	520.0	–	2940.1	–	36.4	–	1004.2	–	16.1	–
	5	524.8	96.1 (1.4%)	2944.9	96.6 (1.3%)	41.2	96.2 (1.3%)	1009.0	95.1 (1.4%)	20.9	95.3 (1.5%)
	10	529.7	97.2 (1.3%)	2949.9	97.5 (1.2%)	46.1	97.4 (1.2%)	1013.8	95.9 (1.3%)	25.8	96.7 (1.4%)
Rice	zero	480.6	–	2150.3	–	14.5	–	520.9	–	3.4	–
	5	485.5	97.8 (1.3%)	2155.2	97.2 (1.3%)	19.4	98.3 (1.1%)	525.8	97.3 (1.2%)	8.3	98.1 (1.2%)
	10	490.5	98.6 (1.2%)	2160.2	98.5 (1.1%)	24.4	99.1 (1.0%)	530.8	98.4 (1.1%)	13.4	99.5 (1.0%)
Corn	zero	123.3	–	2861.3	–	82.7	–	1015.3	–	N. D.^a^	–
	5	128.3	100.6 (1.1%)	2866.4	101.3 (1.2%)	87.7	100.9 (1.1%)	1020.4	101.4 (1.2%)	5.1	101.6 (1.2%)
	10	133.3	99.7 (1.0%)	2871.4	100.9 (1.0%)	92.7	99.8 (1.0%)	1025.4	100.7 (1.1%)	10.0	100.4 (1.1%)
Honey	zero	320.9	–	1930.7	–	15.2	–	367.90	–	N. D.	–
	5	325.8	98.0 (1.3%)	1935.7	98.2 (1.2%)	20.1	97.8 (1.2%)	372.80	98.1 (1.1%)	4.9	98.6 (1.2%)
	10	330.8	98.7 (1.2%)	1940.7	99.5 (1.0%)	25.1	98.6 (1.1%)	377.83	99.3 (1.0%)	9.9	99.7 (1.0%)
Hen egg	zero	31.8	–	1219.8	–	25.6	–	80.70	–	2.7	–
	5	36.7	98.8 (1.2%)	1224.7	98.1 (1.2%)	30.5	98.0 (1.2%)	85.61	98.2 (1.1%)	7.6	98.5 (1.2%)
	10	41.8	99.7 (1.0%)	1229.8	99.5 (1.0%)	35.5	98.9 (1.0%)	90.64	99.4 (1.0%)	12.6	99.3 (1.0%)

a Not Detected.

The accuracy and reliability of developed method were evaluated by analysis of certified reference materials (CRMs; SRM-1974c and NIST-1643f) to approve validity of the analytical results. As shown in Table S5, the measured concentrations exhibited excellent comply with certified and non-certified values, confirming accuracy of the method.

### 3.8 Greenness assessment

**Fig. 6:**
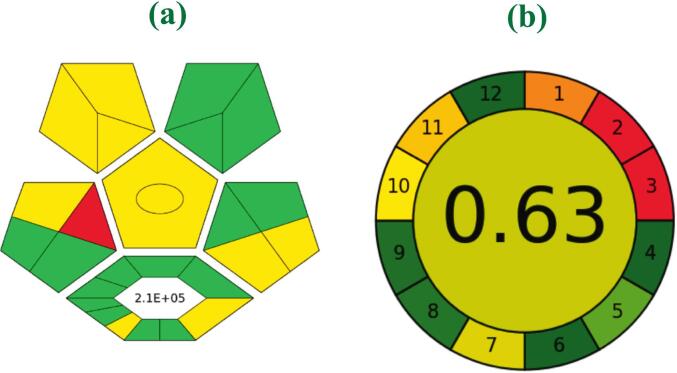
(a) GAPI for greenness assessment, and (b) Agree graph for proposed UA-d-μ-SPE method using 12 environmental indicators.

The environmental sustainability of the UA-D-μ-SPE technique coupled with ICP-OES for extracting heavy metal ions, namely Ni (II), Cu (II), Cd (II), Pb (II), and Hg (II), was rigorously assessed with respect to the twelve principles of green analytical chemistry. Such a multidimensional evaluation considered such criteria as reagent toxicity, waste minimization, energy efficiency, material renewability, biodegradability, and overall method sustainability. The findings, visually summarized in Fig. 6a, employ a circular chart where a central composite score of 0.63 (on a 0–1 scale) signifies a generally positive ecological profile for this analytical procedure. Most evaluated indicators reside in the green sector, vouchsafing method's strengths in multi-elemental analysis, reduced solvent toxicity, and low energy demand; meanwhile, moderate assessment in parameters like waste production, degree of automation, and extent of miniaturization are reflected in lighter green hues.

Further methodological greenness was interrogated using the Green Analytical Procedure Index (GAPI), which visualizes the eco-friendliness of all protocol stages, from sampling and sample preparation to instrumentation and waste management, through a pentagrammatic chart. As depicted in Fig. 6b, most attributes of the technique conform well to green chemistry ideals; the principal shortcoming is related solely to its off-line sample handling. Collectively, these quantitative and qualitative assessments establish that the developed UA-d-μ-SPE–ICP-OES method is largely in alignment with sustainability criteria and represents a significant advancement toward greener analytical practices.

### 3.9 Comparison with other studies

To evaluate the capability of devised procedure for preconcentration and determination of the target heavy metal ions, its figures of merits were compared with those of various sample preparation techniques (Table 4), including SPE, SPME, LPE, and LPME, as well as different analytical detection instruments employed for quantification of Ni (II), Cu (II), Cd (II), Pb (II), and Hg (II) ions. The comparative assessment clearly demonstrated the superior performance of proposed method, particularly in terms of lower limits of detection (LOD), reduced relative standard deviation (RSD), wide linear dynamic range (LDR), and suitable enrichment factor (EF) highlighting its high proceeds to the previously reported approaches.

**Table 4:** Comparative data for determination of Ni (II), Cu (II), Cd (II), Pb (II), and Hg (II).

Extraction method	Analyte	EF (PF)	LOD (ng mL^−1^)	LDR (ng mL^−1^)	RSD%	References
MDSPE-ICP-OES^a^	Ni (II)	6.6	1.10	5.0–500.0	7	(Abellán-Martín et al., 2023)
	Cu (II)	7.8	0.10	0.5–500.0	6	
	Cd (II)	10.0	0.12	0.5–500.0	6	
	Pb (II)	7.9	1.10	5.0–500.0	8	
	Hg (II)	12.0	0.13	0.5–500.0	7	
MSPE-GF-AAS^b^	Cu (II)	–	1.51	5–2000	6.6	(Huang et al., 2019)
	Cd (II)		1.08	5–2000	5.6	
	Pb (II)		1.28	5–2000	5.3	
In-tip SPME-CVAAS^c^	Hg (II)	120	0.02	0.2–50.0	3.1	(Rezaei Kahkha et al., 2017)
Chelating-ICP-OES	Ni (II)	80	2.60	100–2000	1.3	(Feist & Mikula, 2014)
	Cu (II)		1.60	200–4000	1.4	
	Cd (II)		0.17	20–400	3.0	
	Pb (II)		0.92	100–2000	2.1	
SPE-FAAS^d^	Ni (II)	100	0.55	–	<5	(Gouda, 2014)
	Cu (II)		0.30			
	Cd (II)		0.65			
	Pb (II)		0.60			
DES-AA-LL- ELLME-FAAS^e^	Ni (II)	67	0.99	3.3–250.0	2.9	(Ezoddin et al., 2018)
	Cu (II)	69	0.36	1.2–100.0	1.8	
	Cd (II)	68	0.31	1.0–100.0	2.1	
	Pb (II)	67	0.83	2.7–200.0	3.1	
UA-MR-IL-DLLME-GFAAF^f^	Cd (II)	48	0.10	0.3–20.0	3.4	(Yao et al., 2017)
	Pb (II)	48	0.15	0.5–40.0	2.8	
TDLLME-FAAS^g^	Cd (II)	80.1	0.056	2–50	3.01	(Bilal et al., 2017)
	Pb (II)	78.2	0.560	2–50	3.12	
CO2-EA- DμSPE-FAAS^h^	Ni (II)	22.2	1.4	5–550	5.5	(Rajabi et al., 2020)
	Cd (II)	20.6	0.9	3–100	2.5	
	Pb (II)	21.0	2.1	7–750	5.3	
DES-TDLLME-ICP- OES^i^	Cd (II)	32.31	0.06	0.2–20.0	3.16–6.61	(Qezelje et al., 2025)
	Pb (II)	32.86	0.64	2.2–200.0		
	Hg (II)	31.73	0.15	0.5–50.0		
MSPE-ICP-OES	Hg (II)	3	0.05	0.2–1000.0	1.6	(García-Mesa et al., 2019)
UA-d-μ-SPE-ICP-OES	Ni (II)	52.26	0.15	0.50–150.0	3.0	This work
	Cu (II)	52.74	0.10	0.35–120.0	2.9	
	Cd (II)	52.36	0.04	0.15–80.0	2.8	
	Pb (II)	52.54	0.30	1.00–200.0	3.0	
	Hg (II)	52.14	0.09	0.30–100.0	3.1	

a Magnetic dispersive SPE coupled with ICP/OES.

b Magnetic SPE coupled with graphite furnace AAS.

c Inserted into a pipette-tip solid-phase micro-extraction coupled with Cold Vapor Atomic Absorption Spectroscopy.

d SPE coupled with flame AAS.

e Deep eutectic solvent-based air assisted ligand less emulsification LLME coupled with flame AAS.

f Ultrasound-assisted magnetic retrieval-linked ionic liquid D-LLME coupled with graphite furnace AAS.

g Tunable dispersive liquid-liquid micro extraction coupled with flame atomic absorption spectrometry.

h CO_2_-effervescence assisted dispersive micro SPE coupled with flame AAS.

i Deep eutectic solvent- Tandem dispersive liquid-liquid micro-extraction coupled with inductively coupled plasma optical emission spectrometry.

## 4 Conclusion

In summary, Chlorella/ZnO/ZnFe₂O₄ nanocomposite-supported UA-D-μ-SPE method coupled with ICP-OES detection offers an environmentally advanced, robust determination of heavy metals at trace levels in food samples of complex matrices. This novel approach excelled because a highly bioactive, magnetically responsive composite was integrated into a method that combined algal biomass adsorption affinity with functional metal oxides' separation efficiency. The analytical results confirmed impressive capability of this method: under optimized conditions, the limits of detection for Ni (II), Cu (II), Cd (II), Pb (II), and Hg (II) were equal to or lower than 0.3 ng mL^−1^, enrichment factors more than or equal to 52.14, and linearity ranging from 0.15 to 200.00 ng mL^−1^. The procedure maintained exceptional precision as reflected by RSDs ≤3.1% in replicate measurements (*n* = 5). Application to various real samples of grains, vegetables, seafood, and animal-derived products demonstrated consistently high extraction recoveries with strong selectivity to analytes, even in complex matrices. These results validate the practical applicability of the method for routine food safety monitoring and regulatory compliance. The merit of the technique is further supported by the greenness profile through twelve metrics and the GAPI tool. Most indicators fell within the green zone, indicating excellent compliance in solvent toxicity, energy usage, and multi-analyte capability, with only minor areas for improvement in waste generation and miniaturization. Similarly, the AGREE assessment confirmed the method's eco-friendly design, supported by a central score of 0.63 (scale 0–1), demonstrates effective harmonization of analytical and environmental objectives. The method addresses growing demands for sustainable analytical procedures while maintaining superior performance standards. The developed nanocomposite shows potential for broader applications beyond food analysis, including environmental monitoring and food processing applications. Future work could focus on further miniaturization and automation to enhance throughput and reduce operational costs. Altogether, the developed UA-d-μ-SPE–ICP-OES strategy sets a high benchmark for precision, sensitivity, and ecological compatibility in food safety analyses. It provides a scalable template for future advancements, in green analytical chemistry.

## CRediT authorship contribution statement

**Hamidreza Haghgoo Qezelje:** Writing – review & editing, Writing – original draft, Validation, Software, Investigation, Funding acquisition, Formal analysis, Data curation, Conceptualization. **Maryam Rajabi:** Writing – review & editing, Supervision, Project administration, Methodology, Formal analysis. **Fatemeh Darabi:** Writing – review & editing, Software. **Yasaman Sedaghat:** Writing – original draft, Software, Investigation, Data curation. **Amir Sajad Soleimani Kia:** Writing – original draft, Data curation. **Alireza Asghari:** Writing – original draft, Validation, Conceptualization. **Felipe de J. Silerio-Vázquez:** Writing – review & editing, Writing – original draft, Software, Investigation. **Khalil Ahmad:** Writing – review & editing, Writing – original draft. **Ahmad Hosseini-Bandegharaei:** Writing – review & editing, Writing – original draft, Validation, Software, Project administration, Investigation.

## Declaration of competing interest

The authors declare that they have no known competing financial interests or personal relationships that could have appeared to influence the work reported in this paper.

## Data Availability

Data will be made available on request.

## References

[bb0005] Abellán-Martín S.J., Villalgordo-Hernández D., Aguirre M.Á., Ramos-Fernández E.V., Narciso J., Canals A. (2023). Enhancing trace metal extraction from wastewater: Magnetic activated carbon as a high-performance sorbent for inductively coupled plasma optical emission spectrometry analysis. Separations.

[bb0010] Al-Arjan W.S. (2022). Zinc oxide nanoparticles and their application in adsorption of toxic dye from aqueous solution. Polymers.

[bb0015] Al-Nassar S.I., Hussein F.I. (2019). The effect of laser pulse energy on ZnO nanoparticles formation by liquid phase pulsed laser ablation. Journal of Materials Research and Technology.

[bb0020] Alshehri A., Alharbi L., Wani A.A., Malik M.A. (2024). Biogenic punica granatum flower extract assisted ZnFe₂O₄ and ZnFe₂O₄–cu composites for excellent photocatalytic degradation of RhB dye. Toxics.

[bb0025] Arghavani-Beydokhti S., Darabi F., Rajabi M., Hosseini-Bandegharaei A., Asghari A., Bayuo J., Noroozi F. (2026). Ultrasound-assisted extraction and sensitive determination of famotidine and ranitidine in biological samples using magnetic spirulina–clinoptilolite nanocomposite. Microchemical Journal.

[bb0030] Azzouz A., Kailasa S.K., Lee S.S., Rascón A.J., Ballesteros E., Zhang M., Kim K.H. (2018). Review of nanomaterials as sorbents in solid-phase extraction for environmental samples. TrAC Trends in Analytical Chemistry.

[bb0035] Bilal M., Kazi T.G., Afridi H.I., Ali J., Baig J.A., Arain M.B., Khan M. (2017). A new tunable dispersive liquid–liquid microextraction method developed for the simultaneous preconcentration of lead and cadmium from lake water: A multivariate study. Spectrochimica Acta Part A: Molecular and Biomolecular Spectroscopy.

[bb0040] Bozorgzadeh E., Pasdaran A., Ebrahimi-Najafabadi H. (2021). Determination of toxic heavy metals in fish samples using dispersive micro solid phase extraction combined with inductively coupled plasma optical emission spectroscopy. Food Chemistry.

[bb0045] Bulut V.N., Gundogdu A., Duran C., Senturk H.B., Soylak M., Elci L., Tufekci M. (2007). A multi-element solid-phase extraction method for trace metals determination in environmental samples on amberlite XAD-2000. Journal of Hazardous Materials.

[bb0050] Cao Y., Qin J., Su Z., Cai L., Fang G., Wang S. (2023). Novel poly(N-methacryloyl-L-alanine acid) grafted chitosan microspheres based solid-phase extraction coupled with ICP-MS for simultaneous detection of trace metal elements in food. Food Chemistry*: X*.

[bb0055] Ebrahimi-Najafabadi H., Pasdaran A., Bezenjani R.R., Bozorgzadeh E. (2019). Determination of toxic heavy metals in rice samples using ultrasound-assisted emulsification microextraction combined with inductively coupled plasma optical emission spectroscopy. Food Chemistry.

[bb0060] Elci L., Soylak M., Uzun A., Büyükpatır E., Doğan M. (2000). Determination of trace impurities in some nickel compounds by flame atomic absorption spectrometry after solid phase extraction using amberlite XAD-16 resin. Fresenius’ Journal of Analytical Chemistry.

[bb0065] Elsaesser T., Schauss J., Kundu A., Fingerhut B.P. (2021). Phosphate vibrations probe electric fields in hydrated biomolecules: Spectroscopy, dynamics, and interactions. The Journal of Physical Chemistry B.

[bb0070] Ezoddin M., Lamei N., Siami F., Abdi K., Karimi M.A. (2018). Deep eutectic solvent based air assisted ligandless emulsification liquid–liquid microextraction for preconcentration of some heavy metals in biological and environmental samples. Bulletin of Environmental Contamination and Toxicology.

[bb0075] Fang W.Q., Zhang B., Yang H.G. (2013). High yield synthesis and magnetic properties of ZnFe₂O₄ single crystal nanocubes in aqueous solution. Journal of Alloys and Compounds.

[bb0080] Farahani S., Eshghi N., Abbasi A., Karimi F., Shiri Malekabad E., Rezaei M. (2015). Determination of heavy metals in albumen of hen eggs from the Markazi province (Iran) using ICP-OES technique. Toxin Reviews.

[bb0085] Feist B., Mikula B. (2014). Preconcentration of heavy metals on activated carbon and their determination in fruits by inductively coupled plasma optical emission spectrometry. Food Chemistry.

[bb0090] Feist B., Mikula B., Pytlakowska K., Puzio B., Buhl F. (2008). Determination of heavy metals by ICP-OES and F-AAS after preconcentration with 2,2′-bipyridyl and erythrosine. Journal of Hazardous Materials.

[bb0095] García-Mesa J.C., Leal P.M., Guerrero M.L., Alonso E.V. (2019). Simultaneous determination of noble metals, Sb and Hg by magnetic solid-phase extraction on-line ICP-OES based on a new functionalized magnetic graphene oxide. Microchemical Journal.

[bb0100] Genchi G., Carocci A., Lauria G., Sinicropi M.S., Catalano A. (2020). Nickel: Human health and environmental toxicology. International Journal of Environmental Research and Public Health.

[bb0105] Ghorban M., Ojaghzadeh Khalil Abad M., Keshavarzi M. (2025). A novel MOF-on-MOF composite versus its MOF shell: A comparative sorbent study for dispersive micro-solid phase extraction of pesticides in food samples. Food Chemistry*: X*.

[bb0110] Gouda A.A. (2014). Solid-phase extraction using multiwalled carbon nanotubes and quinalizarin for preconcentration and determination of trace amounts of some heavy metals in food, water and environmental samples. International Journal of Environmental Analytical Chemistry.

[bb0115] Haghgoo Qezelje H., Rajabi M., Shirmahi A., Ghanbari-Adivi S., Hosseini-Bandegharaei A., Asghari A., Memarian F. (2025). One-pot/one-step synthesis of bi₂O₃/ZnO/Pd nanocomposite for preconcentration and determination of some heavy metal ions in different samples. International Journal of Environmental Analytical Chemistry.

[bb0120] Huang Y., Peng J., Huang X. (2019). Allylthiourea functionalized magnetic adsorbent for the extraction of cadmium, copper and lead ions prior to their determination by atomic absorption spectrometry. Microchimica Acta.

[bb0125] Joo G., Lee W., Choi Y. (2021). Heavy metal adsorption capacity of powdered chlorella vulgaris biosorbent: Effect of chemical modification and growth media. Environmental Science and Pollution Research.

[bb0130] Khatoon N., Ali S., Hussain A., Huang J., Yu Z., Liu H. (2024). Human health risks assessment of toxic metals via water, food, and soil: A case study of northern areas (Ghizer and Gilgit) of Pakistan. Results in Engineering.

[bb0135] Qezelje H.H., Rajabi M., Prakash C., Hosseini-Bandegharaei A., Adivi S.G., Asghari A. (2025). Simultaneous preconcentration and measurement of ultra-trace Cd (II), Pb (II), and Hg (II) from food samples using deep eutectic solvents in a green approach and employing ICP-OES. Journal of Food Composition and Analysis.

[bb0140] Rajabi M., Mollakazemi Z., Hemmati M., Arghavani-Beydokhti S. (2020). CO₂-effervescence assisted dispersive micro solid-phase extraction based on a magnetic layered double hydroxide modified with polyaniline and a surfactant for efficient preconcentration of heavy metals in cosmetic samples. Analytical Methods.

[bb0145] Rasin P., Ashwathi A.V., Basheer S.M., Haribabu J., Santibanez J.F., Garrote C.A., Mangalaraja R.V. (2025). Exposure to cadmium and its impacts on human health: A short review. Journal of Hazardous Materials Advances.

[bb0150] Rezaei Kahkha M.R., Daliran S., Oveisi A.R., Kaykhaii M., Sepehri Z. (2017). The mesoporous porphyrinic zirconium metal–organic framework for pipette-tip solid-phase extraction of mercury from fish samples followed by cold vapor atomic absorption spectrometric determination. Food Analytical Methods.

[bb0155] Sadegh N., Asfaram A., Javadian H., Haddadi H., Sharifpour E. (2021). Ultrasound-assisted solid phase microextraction-HPLC method based on Fe_3_O_4_@SiO_2_-NH_2_-molecularly imprinted polymer magnetic nano-sorbent for rapid and efficient extraction of harmaline from *Peganum harmala* extract. Journal of Chromatography B.

[bb0160] Sahoo P., Choudhary P., Laha S.S., Dixit A., Mefford O.T. (2023). Recent advances in zinc ferrite (ZnFe₂O₄) based nanostructures for magnetic hyperthermia applications. Chemical Communications.

[bb0165] Sen I., Shandil A., Shrivastava V.S. (2011). Study for determination of heavy metals in fish species of the river yamuna (Delhi) by inductively coupled plasma optical emission spectroscopy (ICP OES). Journal of Advanced Applied Science Research.

[bb0170] Shirmahi A., Rajabi M., Qezelje H.H., Youssefian H., Adivi S.G., Zolfaghari S., Asghari A. (2025). ZnO/chitin/Fe3O4 nanocomposite as an efficient adsorbent for preconcentration and trace determination of non-steroidal anti-inflammatory drugs. Results in Chemistry.

[bb0175] Smith B.C. (2018). The carbonyl group, Part V: Carboxylates—Coming clean. Spectroscopy.

[bb0180] Sukoviene A., Ali S., Jagminas A., Ramanavicius S. (2025). Magnetic cobalt and other types of ferrite nanoparticles: Synthesis aspects and novel strategies for application in wastewater treatment. Applied Sciences.

[bb0185] Telloli C., Cicconi F., Manzi E., Borgognoni F., Salvi S., Iapalucci M.C., Rizzo A. (2024). Multi-elemental analysis of commercial wheat flours by ICP-MS triple quadrupole in function of the milling degree. Food Chemistry.

[bb0190] Tibebe D., Hussen M., Mulugeta M., Yenealem D., Moges Z., Gedefaw M., Kassa Y. (2022). Assessment of selected heavy metals in honey samples using flame atomic absorption spectroscopy (FAAS), Ethiopia. BMC Chemistry.

[bb0195] Witt B., Friese S., Walther V., Ebert F., Bornhorst J., Schwerdtle T. (2025). Cellular mechanisms of copper neurotoxicity in human, differentiated neurons. Archives of Toxicology.

[bb0200] World Health Organization (WHO) (2021). http://0.0.7.233.

[bb0205] Yao L., Wang X., Liu H., Lin C., Pang L., Yang J., Zeng Q. (2017). Optimization of ultrasound-assisted magnetic retrieval-linked ionic liquid dispersive liquid–liquid microextraction for the determination of cadmium and lead in water samples by graphite furnace atomic absorption spectrometry. Journal of Industrial and Engineering Chemistry.

[bb0210] Yap C.K., Al-Mutairi K.A. (2023). Biomonitoring–health risk nexus of potentially toxic metals on *Cerithidea obtusa*: A biomonitoring study from Peninsular Malaysia. Foods.

[bb0215] Zhao T., Han X., He L., Jia Y., Yu R.C. (2022). Fourier transform infrared spectrometry detection of phaeodactylum tricornutum biomacromolecules in response to environmental changes. ACS Omega.

