# Supplementary material for: Synthesis of a Chlorella/ZnO/ZnFe2O4 green nanocomposite for preconcentration of heavy metals in food samples via ICP-OES detection
Source: Food Chem X. 2026 Apr 24;36:103852. doi: 10.1016/j.fochx.2026.103852 (PMC13141770; doi:10.1016/j.fochx.2026.103852)
Supplement: Supplementary file 1 — Supplementary material [file mmc1.docx]

***Supplementary Data***

Hamidreza Haghgoo Qezelje^a^, Maryam Rajabi^a,*^, Fatemeh Darabi^a^, Yasaman Sedaghat^a^, Amir Sajad Soleimani Kia^b^, Alireza Asghari^a^, Felipe de J. Silerio-Vázquez^c^, Khalil Ahmad^d^, Ahmad Hosseini-Bandegharaei^a,e^,

*^a^Department of Chemistry, Semnan University, Semnan, Iran*

*^b^Department of Chemistry, University of Kashan, Kashan 87317-53153, Iran*

*^c^CIIDIR-Durango, Instituto Politécnico Nacional, Calle Sigma 119, Fraccionamiento 20 de Noviembre II, C. P. 34220, Durango, México*

*^d^Department of Chemistry, Emerson University Multan (EUM), Multan-60000, Pakistan*

*^e^* *Scientific Research Center, Al-Ayen Iraqi University (AUIQ), Nasiriyah, 64001, Thi-Qar, Iraq*

^٭^Corresponding author: Email [mrajabi@semnan.ac.ir](mailto:mrajabi@semnan.ac.ir) & [marajabi@gmail.com](mailto:marajabi@gmail.com) (M. Rajabi); Phone: +98-23-33383193; Fax: +98-231-33654110

**Table S1.** A CCD-based 5-level design on one-block and 3 principal factors for UA-d-μ-SPE procedure in sorption of studying heavy metals. Conditions: 100 mL of aqueous solution containing Ni (II), Pb (II),
Cu (II), and Hg (II) ions (each 20 ng mL⁻¹) and Cd (II) ions (10 ng mL⁻¹); 2 mL of hydrochloric acid (0.3 mol L⁻¹) as eluting solvent; and desorption time 3 min.

| Independent variables | Ranges and levels | | | | |
| --- | --- | --- | --- | --- | --- |
|  | -α | -1 | 0 | +1 | +α |
| Sample Solution pH (A) | 2 | 3.62 | 6 | 8.38 | 10 |
| Sorbent Dosage (mg) (B) | 4 | 6.43 | 10 | 13.57 | 16 |
| Extraction Time (min) (C) | 2 | 3.62 | 6 | 8.38 | 10 |

| Run | Factor | | |  | Extraction Recovery (%) | | | | |
| --- | --- | --- | --- | --- | --- | --- | --- | --- | --- |
|  | A | B | C |  | Ni (II) | Cu (II) | Cd (II) | Pb (II) | Hg (II) |
| 1 | 6.0 | 16.0 | 6 |  | 72.4 | 73.8 | 72.6 | 73.2 | 71.6 |
| 2 | 3.6 | 6.4 | 4 |  | 58.8 | 61.7 | 59.9 | 60.1 | 57.8 |
| 3 | 8.4 | 13.6 | 8 |  | 68.2 | 71.3 | 70.1 | 70.3 | 67.5 |
| 4 | 6.0 | 10.0 | 10 |  | 72.4 | 75.1 | 73.3 | 73.9 | 71.1 |
| 5 | 3.6 | 13.6 | 4 |  | 60.1 | 63.2 | 62.1 | 62.5 | 58.9 |
| 6 | 8.4 | 13.6 | 4 |  | 64.7 | 67.6 | 66.1 | 67.2 | 63.9 |
| 7 | 3.6 | 6.4 | 8 |  | 61.9 | 64.7 | 63.5 | 64.5 | 60.8 |
| 8 | 6.0 | 10.0 | 6 |  | 87.2 | 91.3 | 88.7 | 90.3 | 86 |
| 9 | 10.0 | 10.0 | 6 |  | 53.2 | 57.3 | 54.9 | 56.1 | 52.5 |
| 10 | 6.0 | 10.0 | 6 |  | 89.1 | 92.2 | 91.4 | 91.6 | 88.2 |
| 11 | 6.0 | 10.0 | 6 |  | 88.5 | 91.7 | 89.7 | 90.7 | 87.4 |
| 12 | 3.6 | 13.6 | 8 |  | 63.3 | 66 | 65.2 | 65.8 | 62.5 |
| 13 | 6.0 | 4.0 | 6 |  | 65.7 | 68.6 | 66.8 | 67.4 | 64.9 |
| 14 | 8.4 | 6.4 | 4 |  | 60.3 | 63 | 62.2 | 62.9 | 59.2 |
| 15 | 8.4 | 6.4 | 8 |  | 63.2 | 66.3 | 64.7 | 65.3 | 62.3 |
| 16 | 6.0 | 10.0 | 6 |  | 87.9 | 91.1 | 88.6 | 89.9 | 86.6 |
| 17 | 6.0 | 10.0 | 6 |  | 87.6 | 90.8 | 88.5 | 89.5 | 86.4 |
| 18 | 2.0 | 10.0 | 6 |  | 50.5 | 53.2 | 51 | 52.2 | 49.8 |
| 19 | 6.0 | 10.0 | 6 |  | 88.5 | 91.6 | 89.3 | 90.6 | 87.6 |
| 20 | 6.0 | 10.0 | 2 |  | 67.2 | 69.9 | 68.5 | 69.3 | 66 |

**Table S2.** A CCD-based 5-level design on one-block and 3 principal factors for UA-d-μ-SPE procedure in desorption of studying heavy metals. Conditions: 100 mL of aqueous solution containing Ni (II), Pb (II), Cu (II), and Hg (II) ions (each 20 ng mL⁻¹) and Cd (II) ions (10 ng mL⁻¹); sample solution pH adjusted on 6.1; adsorbent amount 10.3 mg; extraction time 6 min; and type of eluting solvent nitric acid.

| Independent variables | Range and levels | | | | |
| --- | --- | --- | --- | --- | --- |
|  | -α | -1 | 0 | +1 | +α |
| Concentration of Eluting Solvent (mol L^-1^) (A) | 0.1 | 0.22 | 0.4 | 0.58 | 0.7 |
| Desorption Time (min) (B) | 1 | 2.22 | 4 | 5.78 | 7 |
| Volume of Eluting Solvent (mL) (C) | 0.9 | 1.33 | 1.95 | 2.57 | 3 |

| Run | Factor | | |  | Extraction Recovery (%) | | | | |
| --- | --- | --- | --- | --- | --- | --- | --- | --- | --- |
|  | A | B | C |  | Ni (II) | Cu (II) | Cd (II) | Pb (II) | Hg (II) |
| 1 | 0.4 | 4 | 0.9 |  | 72.5 | 73.6 | 72.84 | 72.31 | 72.33 |
| 2 | 0.4 | 4 | 1.9 |  | 98.65 | 99.8 | 98.73 | 99.65 | 98.12 |
| 3 | 0.4 | 4 | 1.9 |  | 99.21 | 99.84 | 99.36 | 99.67 | 99.1 |
| 4 | 0.4 | 7 | 1.9 |  | 63.23 | 64.04 | 63.34 | 62.87 | 62.99 |
| 5 | 0.2 | 2 | 1.3 |  | 62.59 | 64.12 | 62.71 | 64.07 | 62.14 |
| 6 | 0.4 | 4 | 1.9 |  | 98.54 | 99.25 | 98.89 | 98.96 | 98.31 |
| 7 | 0.2 | 6 | 2.6 |  | 78.2 | 78.56 | 78.31 | 78.34 | 77.97 |
| 8 | 0.6 | 6 | 1.3 |  | 69.94 | 71.32 | 70.24 | 71.18 | 69.81 |
| 9 | 0.2 | 2 | 2.6 |  | 65.37 | 66.61 | 65.49 | 66.47 | 65.17 |
| 10 | 0.7 | 4 | 1.9 |  | 93.5 | 94.12 | 93.73 | 93.81 | 93.24 |
| 11 | 0.6 | 2 | 1.3 |  | 74.85 | 75.53 | 74.91 | 75.18 | 74.42 |
| 12 | 0.4 | 4 | 3.0 |  | 85.12 | 86.41 | 85.35 | 85.5 | 84.79 |
| 13 | 0.4 | 4 | 1.9 |  | 98.65 | 99.23 | 98.87 | 99.11 | 98.43 |
| 14 | 0.2 | 6 | 1.3 |  | 69.5 | 70.13 | 69.67 | 69.99 | 69.23 |
| 15 | 0.6 | 2 | 2.6 |  | 82.1 | 82.84 | 82.21 | 82.67 | 81.95 |
| 16 | 0.6 | 6 | 2.6 |  | 82.23 | 83.02 | 82.42 | 82.85 | 82.04 |
| 17 | 0.4 | 1 | 1.9 |  | 57 | 57.84 | 57.13 | 57.77 | 56.92 |
| 18 | 0.1 | 4 | 1.9 |  | 78.97 | 79.85 | 79.34 | 79.79 | 78.81 |
| 19 | 0.4 | 4 | 1.9 |  | 98.23 | 99.7 | 98.37 | 99.16 | 98.12 |
| 20 | 0.4 | 4 | 1.9 |  | 99.1 | 99.87 | 99.24 | 99.21 | 98.88 |

**Table S3.** Summarized analyses of variance for CCD in UA-D-μ-SPE method for the studied heavy metal ions in adsorption metal ions.

| Source of variance | Sum of squares | Degree of freedom | Mean squares | F-value | P-value |
| --- | --- | --- | --- | --- | --- |
| Ni (II) |  |  |  |  |  |
| Model | 3169.79 | 9 | 352.20 | 611.93 | < 0.0001 |
| Residual | 5.76 | 10 | 0.5756 |  |  |
| Lack-of-fit | 3.34 | 5 | 0.6684 | 1.38 | 0.3648 |
| Pure error | 2.41 | 5 | 0.4827 |  |  |
| R^2^ = 0.9982 | Adj. R^2^ = 0.9966 | Pred. R^2^ = 0.9909 | Adeq Precision =  70.9613 |  |  |
| Cu (II) |  |  |  |  |  |
| Model | 3226.15 | 9 | 358.46 | 1127.46 | < 0.0001 |
| Residual | 3.18 | 10 | 0.3179 |  |  |
| Lack-of-fit | 1.96 | 5 | 0.3929 | 1.62 | 0.3054 |
| Pure error | 1.22 | 5 | 0.2430 |  |  |
| R^2^ = 0.9990 | Adj. R^2^ = 0.9981 | Pred. R^2^ = 0.9948 | Adeq Precision = 95.7924 |  |  |
| Cd (II) |  |  |  |  |  |
| Model | 3128.69 | 9 | 347.63 | 291.10 | < 0.0001 |
| Residual | 11.94 | 10 | 1.19 |  |  |
| Lack-of-fit | 5.91 | 5 | 1.18 | 0.9794 | 0.5089 |
| Pure error | 6.03 | 5 | 1.21 |  |  |
| R^2^ = 0.9962 | Adj. R^2^ = 0.9928 | Pred. R^2^ = 0.9828 | Adeq Precision =  49.2098 |  |  |
| Pb (II) |  |  |  |  |  |
| Model | 3183.90 | 9 | 353.77 | 494.77 | < 0.0001 |
| Residual | 7.15 | 10 | 0.7150 |  |  |
| Lack-of-fit | 4.52 | 5 | 0.9034 | 1.72 | 0.2841 |
| Pure error | 2.63 | 5 | 0.5267 |  |  |
| R^2^ = 0.9978 | Adj. R^2^ = 0.9957 | Pred. R^2^ = 0.9879 | Adeq Precision = 63.6748 |  |  |
| Hg (II) |  |  |  |  |  |
| Model | 3125.75 | 9 | 347.31 | 504.57 | < 0.0001 |
| Residual | 6.88 | 10 | 0.6883 |  |  |
| Lack-of-fit | 3.41 | 5 | 0.6820 | 0.9817 | 0.5078 |
| Pure error | 3.47 | 5 | 0.6947 |  |  |
| R^2^ = 0.9978 | Adj. R^2^ = 0.9958 | Pred. R^2^ = 0.9901 | Adeq Precision = 64.3780 |  |  |

**Table S4.** Summarized ANOVA for CCD in UA-D-μ-SPE method for studied heavy metal ions in desorption stage.

| Source of variance | Sum of squares | Degrees of freedom | Mean squares | F-value | P-value |
| --- | --- | --- | --- | --- | --- |
| Ni (II) |  |  |  |  |  |
| Model | 3909.80 | 9 | 434.42 | 2495.22 | 3909.80 |
| Residual | 1.74 | 10 | 0.1741 |  |  |
| Lack-of-fit | 1.07 | 5 | 0.2150 | 1.61 | 0.3062 |
| Pure error | 0.6662 | 5 | 0.1332 |  |  |
| R^2^ = 0.9996 | Adj. R^2^ = 0.9992 | Pred. R^2^ = 0.9976 | Adeq Precision =  142.5473 |  |  |
| Cu (II) |  |  |  |  |  |
| Model | 3873.60 | 9 | 430.40 | 2538.43 | < 0.0001 |
| Residual | 1.70 | 10 | 0.1696 |  |  |
| Lack-of-fit | 1.26 | 5 | 0.2514 | 2.87 | 0.1363 |
| Pure error | 0.4385 | 5 | 0.0877 |  |  |
| R^2^ = 0.9996 | Adj. R^2^ = 0.9992 | Pred. R^2^ = 0.9973 | Adeq Precision = 144.2099 |  |  |
| Cd (II) |  |  |  |  |  |
| Model | 3915.98 | 9 | 435.11 | 2120.97 | < 0.0001 |
| Residual | 2.05 | 10 | 0.2051 |  |  |
| Lack-of-fit | 1.41 | 5 | 0.2828 | 2.22 | 0.2012 |
| Pure error | 0.6374 | 5 | 0.1275 |  |  |
| R^2^ = 0.9995 | Adj. R^2^ = 0.9990 | Pred. R^2^ = 0.9970 | Adeq Precision =  131.8012 |  |  |
| Pb (II) |  |  |  |  |  |
| Model | 3882.18 | 9 | 431.35 | 3823.00 | < 0.0001 |
| Residual | 1.13 | 10 | 0.1128 |  |  |
| Lack-of-fit | 0.6898 | 5 | 0.1380 | 1.57 | 0.3156 |
| Pure error | 0.4385 | 5 | 0.0877 |  |  |
| R^2^ = 0.9997 | Adj. R^2^ = 0.9994 | Pred. R^2^ = 0.9983 | Adeq Precision = 157.8878 |  |  |
| Hg (II) |  |  |  |  |  |
| Model | 3907.15 | 9 | 434.13 | 1926.84 | < 0.0001 |
| Residual | 2.25 | 10 | 0.2253 |  |  |
| Lack-of-fit | 1.42 | 5 | 0.2838 | 1.70 | 0.2869 |
| Pure error | 0.8339 | 5 | 0.1668 |  |  |
| R^2^ = 0.9994 | Adj. R^2^ = 0.9989 | Pred. R^2^ = 0.9969 | Adeq Precision = 125.2216 |  |  |

**Table S5.** Analytical results for CRMs.

| Sample |  | Ni (II) | Cu (II) | Cd (II) | Pb (II) | Hg (II) |
| --- | --- | --- | --- | --- | --- | --- |
| SRM-1974c | Certified values (ng mL^-1^) | N. D. | - | - | - | 19.70 ± 0.62 |
|  | Non-Certified value (ng mL^-1^) | - | 1120.0 ± 140.0 | 157.0 ± 4.90 | 800.0 ± 50.0 | - |
|  | Found (ng mL^-1^) | - | 1146.43 ± 132.78 | 158.88 ± 4.53 | 813.12 ± 51.06 | 19.44 ± 0.45 |
|  | Recovery (%) | - | 102.36 | 101.20 | 101.64 | 98.71 |
| NIST-1643f | Certified value (ng mL^-1^) | 59.20 ± 1.400 | 21.44 ± 0.700 | 5.83 ± 0.130 | 18.303 ± 0.081 | N. D. |
|  | Found (ng mL^-1^) | 58.50 ± 1.02 | 21.21 ± 0.66 | 5.68 ± 0.28 | 18.04 ± 0.11 | - |
|  | Recovery (%) | 98.82 | 98.93 | 97.46 | 98.58 | - |


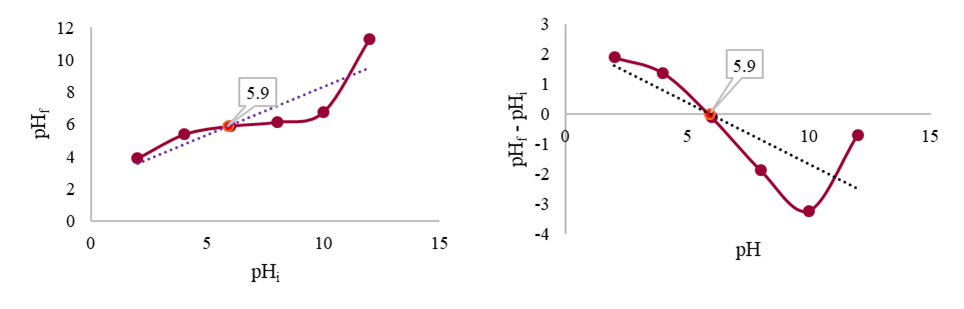


**Fig. S1.** Isoelectric point diagram using (a) simple and (b) drift methods.


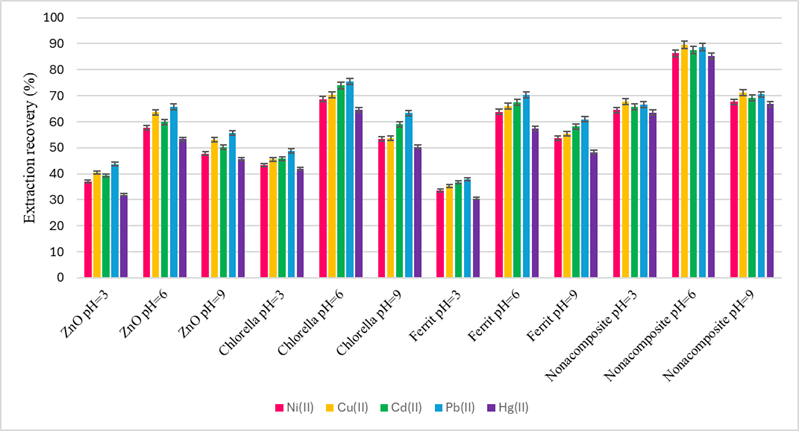


**Fig. S2.** Evaluation of the effect of each component of the synthesized nanocomposite on
extraction recovery (%) of metal ions using UA-D-μ-SPE coupled with ICP-OES detection (Conditions: 100 mL of aqueous solution containing Ni (II), Pb (II), Cu (II), and Hg (II) ions (each 20 ng mL⁻¹) and Cd (II) ions (10 ng mL⁻¹); sorbent dosage 10 mg; extraction time 5 min; 2 mL of hydrochloric acid
(0.3 mol L⁻¹) as eluting solvent; and desorption time 3 min).


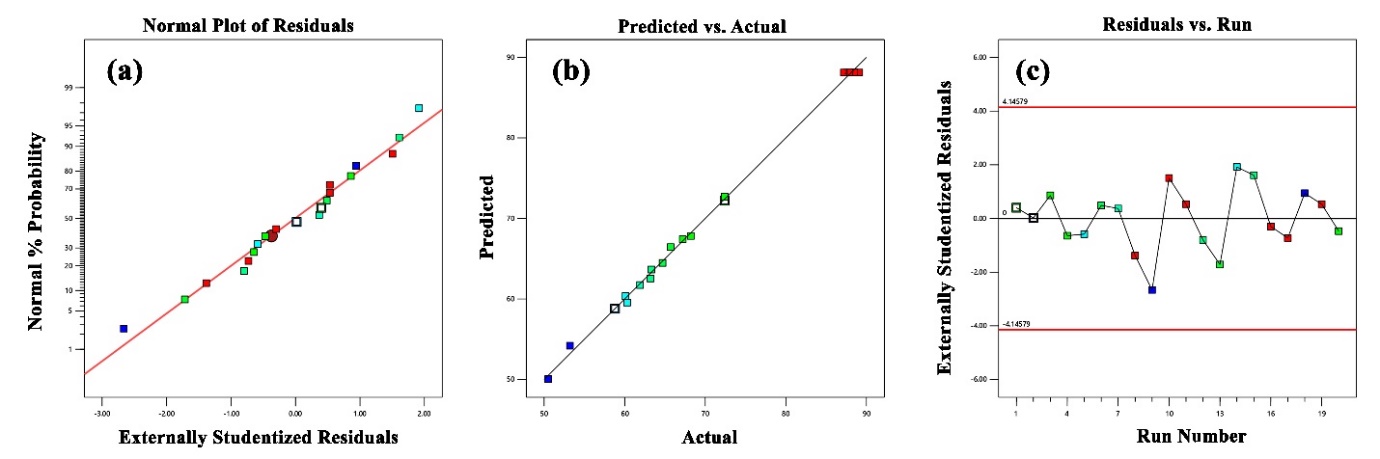


**Fig. S3.** (a) Normal probability plots of externally studentized residuals, (b) predicted versus actual values, and (c) residuals versus run number for Ni (II) in the adsorption stage.


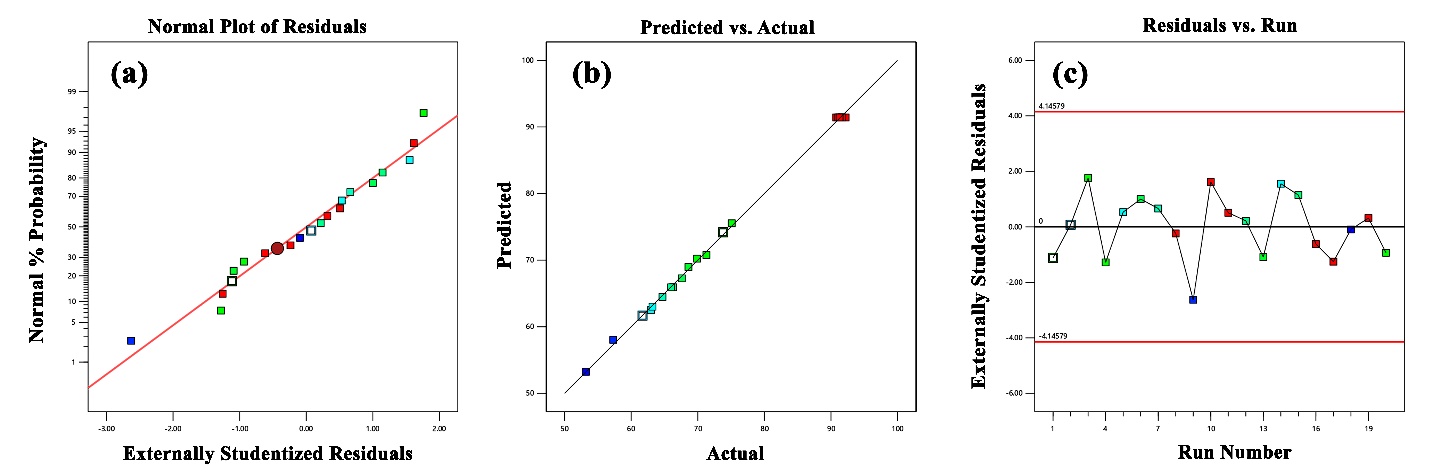


**Fig. S4.** (a) Normal probability plots of externally studentized residuals, (b) predicted versus actual values, and (c) residuals versus run number for Cu (II) in the adsorption stage.


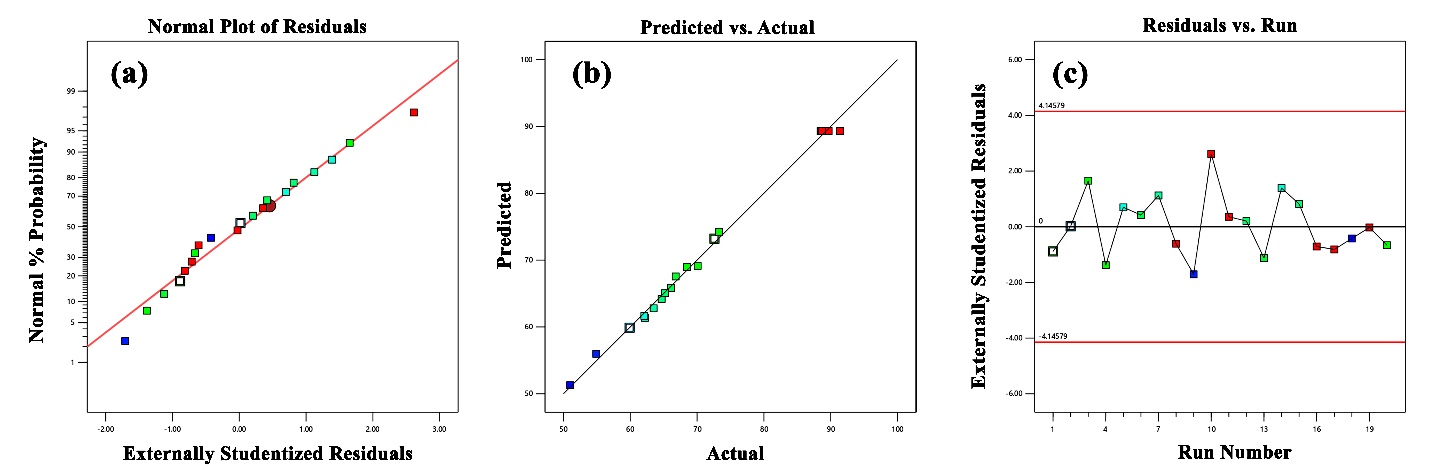


**Fig. S5.** (a) Normal probability plots of externally studentized residuals, (b) predicted versus actual values, and (c) residuals versus run number for Cd (II) in the adsorption stage.


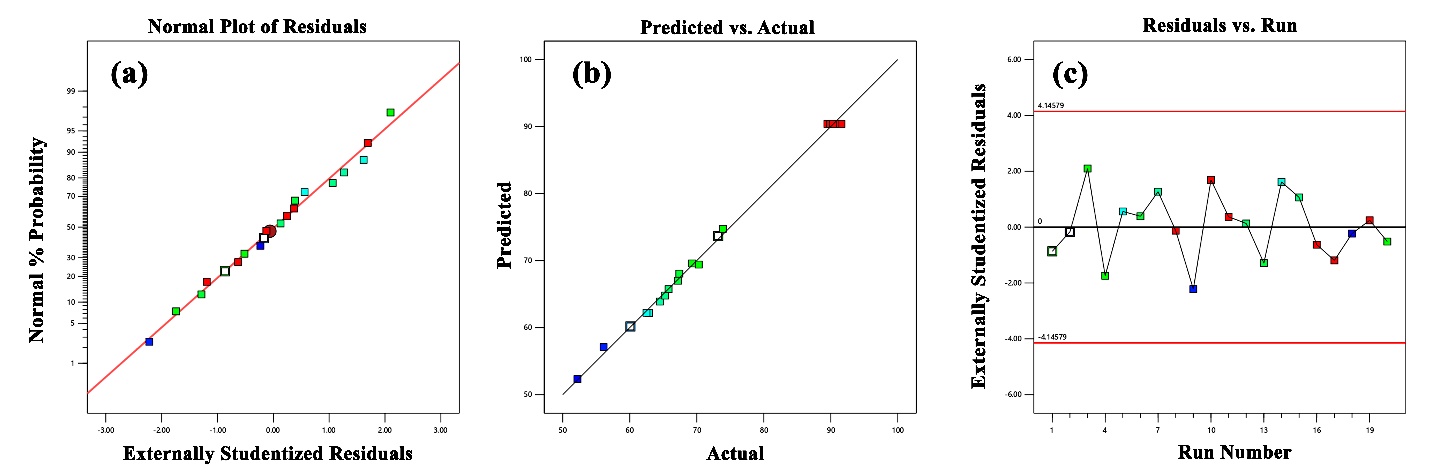


**Fig. S6.** (a) Normal probability plots of externally studentized residuals, (b) predicted versus actual values, and (c) residuals versus run number for Pb (II) in the adsorption stage.


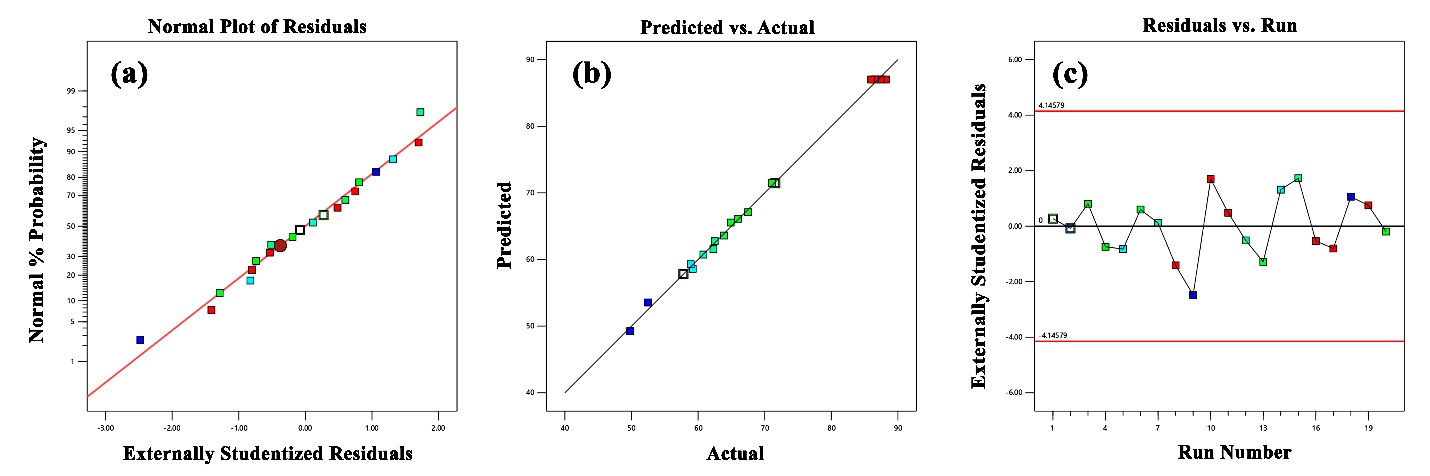


**Fig. S7.** (a) Normal probability plots of externally studentized residuals, (b) predicted versus actual values, and (c) residuals versus run number for Hg (II) in the adsorption stage.


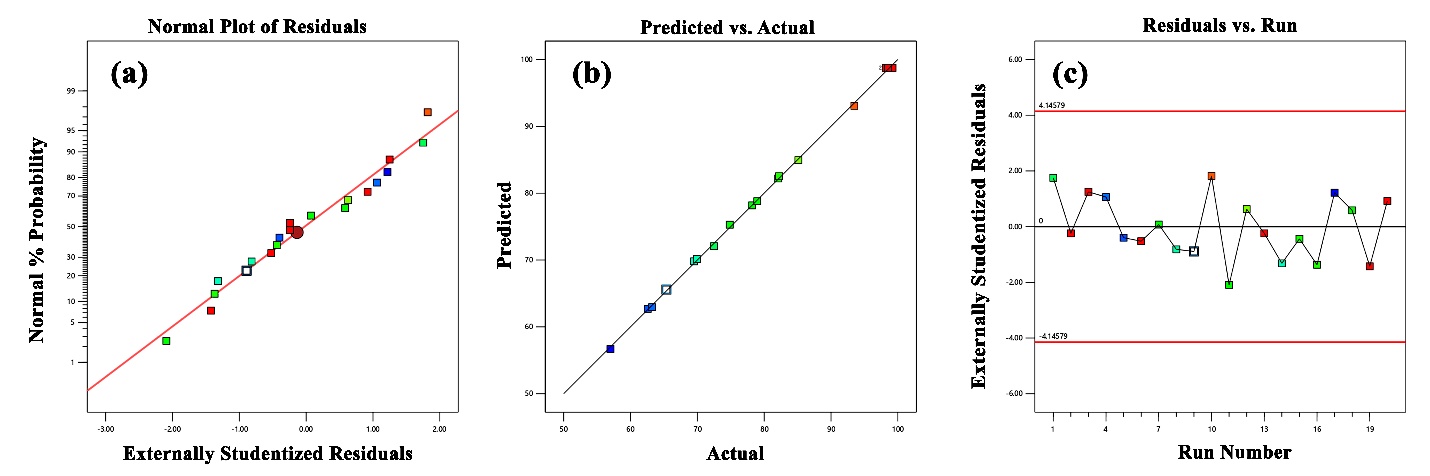


**Fig. S8.** (a) Normal probability plots of externally studentized residuals, (b) predicted versus actual values, and (c) residuals versus run number for Ni (II) in the desorption stage.


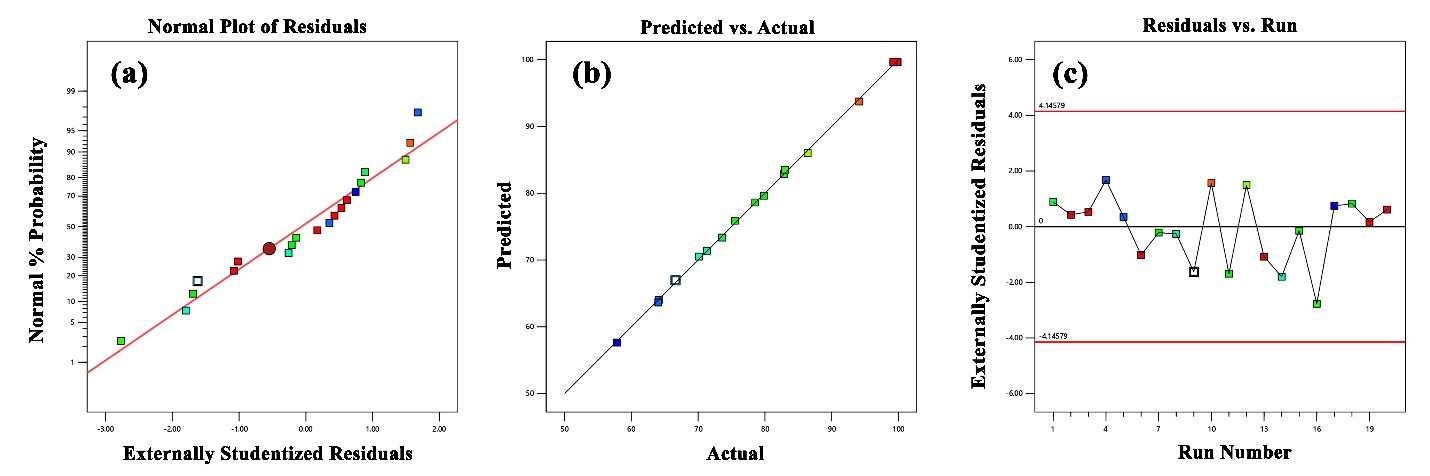


**Fig. S9.** (a) Normal probability plots of externally studentized residuals, (b) predicted versus actual values, and (c) residuals versus run number for Cu (II) in the desorption stage.


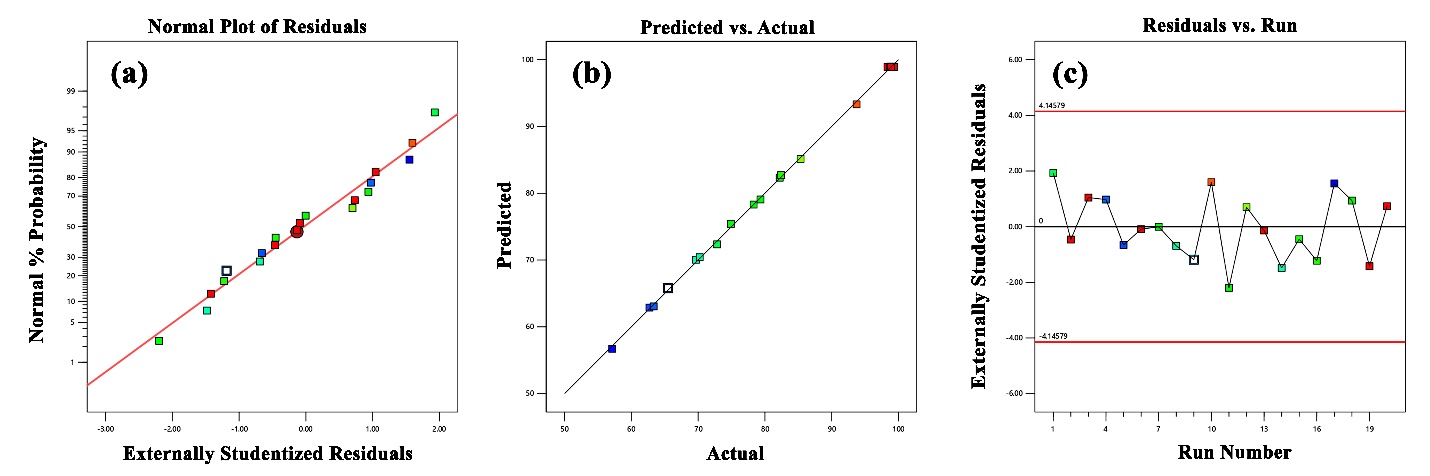


**Fig. S10.** (a) Normal probability plots of externally studentized residuals, (b) predicted versus actual values, and (c) residuals versus run number for Cd (II) in the desorption stage.


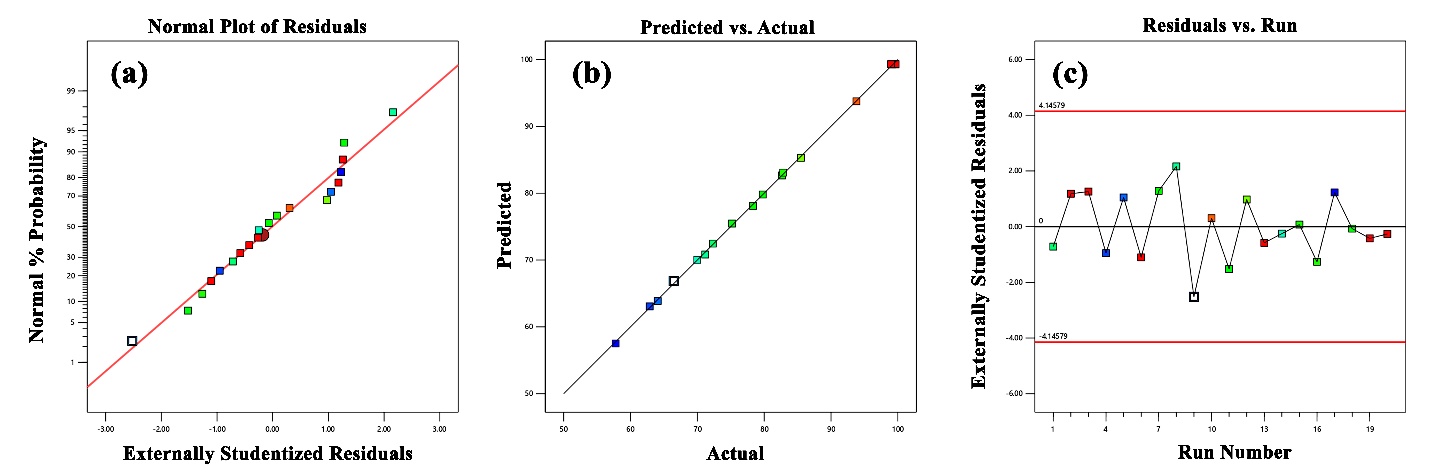


**Fig. S11.** (a) Normal probability plots of externally studentized residuals, (b) predicted versus actual values, and (c) residuals versus run number for Pb (II) in the desorption stage.


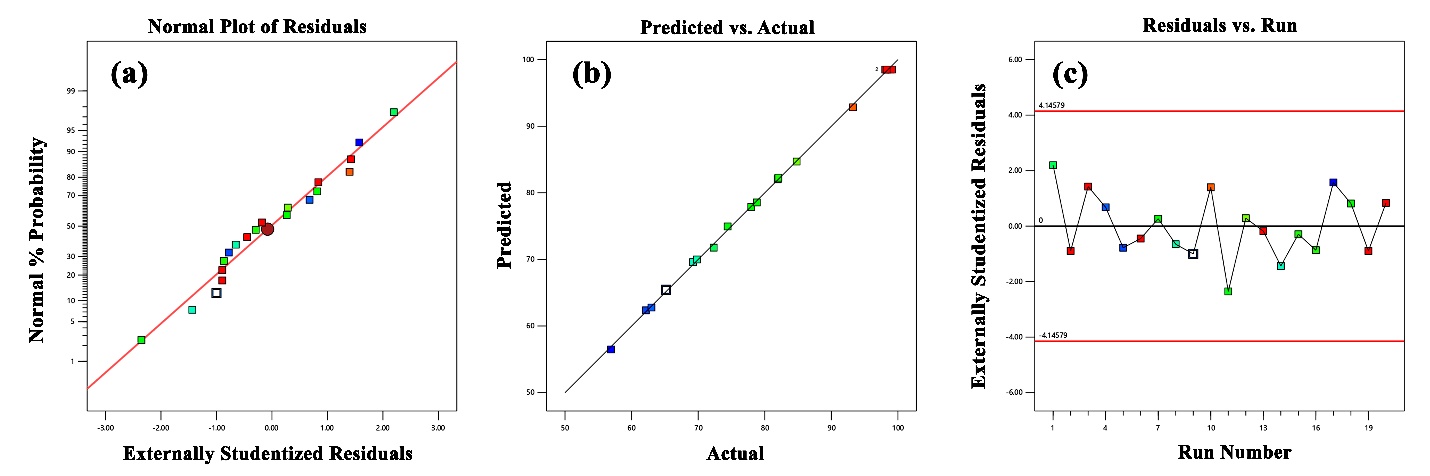


**Fig. S12.** (a) Normal probability plots of externally studentized residuals, (b) predicted versus actual values, and (c) residuals versus run number for Hg (II) in the desorption stage.

**Fig. S13.** Calibration curve for Ni (II) determination.

**Fig. S14.** Calibration curve for Cu (II) determination.

**Fig. S15.** Calibration curve for Cd (II) determination.

**Fig. S16.** Calibration curve for Pb (II) determination.

**Fig. S17.** Calibration curve for Hg (II) determination.

**Fig. S18.** Reusability performance of Chlorella/ZnO/ZnFe_2_O_4_ nanocomposite.

**Fig. S19.** Design Expert optimization plot illustrating the optimal conditions for metal analyte adsorption, maximizing extraction recovery (ER).

**Fig. S20.** Design Expert optimization plot illustrating the optimal conditions for metal analyte desorption, maximizing extraction recovery (ER).
